# Socio-demographic disparities in HER2+ breast cancer trastuzumab receipt: An English population-based study

**DOI:** 10.1158/1055-9965.EPI-24-0144

**Published:** 2024-10-02

**Authors:** Ruth P Norris, Rosie Dew, Alastair Greystoke, Nicola Cresti, Henry Cain, Adam Todd, Linda Sharp

**Affiliations:** 1 Population Health Sciences Institute, Newcastle University, Centre for Cancer, Newcastle-upon-Tyne, United Kingdom; 2 Northern Centre for Cancer Care, Freeman Hospital, Newcastle Hospital Trust, Newcastle-upon-Tyne, United Kingdom; 3 School of Pharmacy, Newcastle University, Newcastle-upon-Tyne, United Kingdom

## Abstract

**Background:**

Socio-demographic disparities in traditional breast cancer treatment receipt in non-publicly funded healthcare systems are well documented. This study investigated trastuzumab receipt by socio-demographic factors within a female, HER2+ breast cancer population in England’s publicly funded National Health Service.

**Methods:**

The English national population-based cancer registry and linked Systemic Anti-Cancer Therapy (SACT) database identified 36,985 women with HER2+ invasive breast cancer diagnosed 01/01/2012–31/12/2017. Multivariable logistic regression determined likelihood of trastuzumab receipt in (i) early and (ii) metastatic disease by deprivation category of area of residence and other socio-demographic characteristics.

**Results:**

Early-stage trastuzumab receipt followed a socio-economic gradient. Women residing in the most deprived areas were 10% less likely to receive trastuzumab (multivariable OR 0.90, (95% CI) 0.83, 0.98) compared to women residing in the least deprived areas. In both early and metastatic disease, trastuzumab receipt was less likely in older women with more comorbidities, ER positive disease, and who were not discussed at a multidisciplinary team meeting.

**Conclusions:**

Despite provision of free at the point of delivery care in England, socio-demographic disparities in early-stage HER2+ trastuzumab receipt occur. Further research determining how inequities contribute to disparities in outcomes is warranted to ensure optimized trastuzumab use for all.

**Impact:**

Fair access to novel cancer treatments regardless of place of residence, socio-demographic characteristics, and/or cancer stage requires prioritization in future cancer improvement policies.

## Introduction

Over the past decades, patients with breast cancer have benefitted from the use of novel, targeted anti-cancer therapies.^1^ The monoclonal antibody (mAb) trastuzumab, targeting the human epidermal growth factor receptor 2 (HER2) in patients with HER2 over-expressed/amplified (HER2+) breast cancer (approximately 20% of breast cancer diagnoses worldwide)^2^ provides a clear example.^3^ Trastuzumab has extended treatment choice beyond traditional treatments (surgery and cytotoxic chemotherapy)^4^ and this has improved prognosis in an aggressive breast cancer sub-type.^5^ While treatment of HER2+ breast cancer is increasingly personalized and evolving,^6,7^ trastuzumab remains a crucial care component, offering women a 33% reduction in breast cancer mortality in early stage disease (ratio of annual death rates=0.67, 95% confidence interval (CI) 0.61-0.73)^8^ and an 18% reduction in overall survival in women with metastatic disease (pooled hazard ratio (HR)=0.82, 95% CI 0.71-0.94).^9^

Breast cancer is subject to socio-economic and socio-demographic disparities. Increased incidence is associated with higher socio-economic status (SES),^10^ whilst higher mortality and reduced survival are linked to women with a lower SES - perhaps in part due to barriers related to treatment access.^11^ Lower SES has historically been associated with reduced receipt of traditional breast cancer treatments, including breast conserving surgery,^12^ neoadjuvant chemotherapy (e.g. anthracyclines and taxanes),^13^ and radiation therapy.^12^ Also access to breast cancer treatment varies by ethnicity, health insurance status, and geographical location.^14,15^ However, this body of research has tended to combine all breast cancer subtypes. It is less clear therefore whether HER2+ specific treatment, in particular targeted and historically high cost treatments such as trastuzumab - which may be hypothesized to be more frequently received by the economically advantaged (i.e. those with private finance and/or insurance) - are also subject to differences in receipt by SES and wider socio-demographic factors.^16^

Real-world evidence documenting socio-economic disparities in novel breast cancer treatment receipt is emerging. For example, a recent meta-analysis concluded that a low SES is associated with lower novel anti-cancer therapy receipt across a range of cancers (including trastuzumab use in HER2+ breast cancer).^17^ Similar findings have subsequently been reported in recent observational studies.^18,19^ In addition, socio-demographic and clinical inequities in trastuzumab receipt are highlighted in an older systematic review of observational studies; receipt was higher in women who were younger, had fewer comorbidities, a higher tumor grade, a larger tumor size, an advanced stage cancer, and a negative hormone receptor status.^20^ However, previous population-based studies have predominantly reported USA data; trastuzumab receipt in a publicly funded healthcare system has seldom been reported.^17^ There is some data from the mixed Chinese^21,22^ and Indian^23^ healthcare systems as well as the publicly funded Canadian and Australian health systems,^24–29^ but these studies are few in number or include comparatively small cohorts of treated patients only (with no denominator populations precluding calculation of odds of receipt). The UK National Health Service (NHS) provides an example of a nationwide publicly funded healthcare system where trastuzumab access is free at the point of delivery to all patients and clinical guidelines are biomarker driven. The only available UK data reported to date has examined trastuzumab initiation in older women in the context of adjuvant chemotherapy receipt.^30^ It therefore remains unclear whether socio-economic disparities in trastuzumab receipt occur in healthcare systems where individual finance and/or insurance are not considered a factor, and whether such inequities are present in patients of all ages, and with both early and metastatic disease.

To investigate, a large population-based observational study was undertaken using NHS data in England. The aim of this study was to determine the association of SES (measured using deprivation category at area of residence) and wider socio-demographic characteristics with receipt of trastuzumab in a (i) stage I-III (early) and; (ii) stage IV (metastatic) HER2+ invasive breast cancer female population using data from a publicly funded healthcare system.

## Materials and Methods

### Study Design and Setting

Population-based data was extracted from the National Cancer Registry Database (NCRD) and Systemic Anti-Cancer Therapy (SACT) dataset in England for all cases of women, of any age, diagnosed with a primary invasive stage I-IV breast tumor (International Classification of Diseases (ICD), Tenth Revision C50.0 – C50.9) between 1^st^ January 2012 and 31^st^ December 2017. Favorable ethical approval was obtained from the Proportionate Review Sub-committee of the West Midlands-Edgbaston Research Ethics Committee on October 16^th^ 2019 (Ref 19/WM/0317). Section 251 of the NHS Act 2006 grants legal permission to register information on diagnosed cancers without the need to seek patient consent. The study was performed in accordance with the Declaration of Helsinki and is reported according to the Strengthening the Reporting of Observational Studies in Epidemiology (STROBE) guidelines (Supplementary Materials and Methods).^31^

### Data Sources & Linkage

The NCRD is a national cancer registry for patients living in England, diagnosed with malignant and pre-malignant neoplasms.^32^ Registry data is compiled into an event-based registration model with patient NHS numbers providing unique identifiers for data linkage.^32^ NCRD data obtained was as follows: deprivation category of area of residence at diagnosis measured using quintile rank of the incomes domain of the Index of Multiple Deprivation (IMD), sex, age, year of diagnosis, ethnicity, rural/urban residence, government region,^33^ stage at diagnosis (tumor, nodes, and metastasis (TNM) summary stage),^34^ grade, HER2 status, estrogen receptor (ER) status, presence of multiple tumors, number of comorbidities, discussion at a multidisciplinary team meeting (MDT), and receipt of cancer directed surgery and/or chemotherapy within six months of diagnosis.

SACT is a relatively new resource, capturing drug level information on routinely administered SACT (e.g. standard chemotherapy, targeted therapy, immunotherapy, targeted biologicals, and modifying supportive therapies) in secondary and tertiary NHS providers in England.^35^ Data collection started in April 2012 and by April 2014, monthly NHS hospital Trust submissions were mandated.^36,37^ The SACT data guides treatment delivery and informs the National Institute for Health and Care Excellence’s (NICE) drug funding decisions, though other uses (e.g. audits, research provision, drug monitoring, and clinical trial data follow up) exist.^35,38^ SACT data, linked to cancer registrations, provided information on trastuzumab receipt.

### Study Population

The population of interest were women diagnosed with stage I-IV HER2+ breast cancer (defined as 3+HER2 staining on immunohistochemistry (IHC) or HER2 amplification using in-situ hybridization if 2+ on IHC). To manage instances of multiple primary breast tumors registrations in patients, a hierarchy determined which tumor record to retain for analysis: (i) earliest diagnosis; (ii) most advanced stage at diagnosis; (iii) most specific ICD code (ICD C50.0-C50.8) or; (iv) first tumor entry. Males were excluded as male breast cancer is rare (n=1,815). Further exclusions included tumors with negative or unknown HER2 status (n=221,299) and tumors with a stage at diagnosis of 0 and/or unknown stage (n=3,293). This left an analytical cohort of 36,985 patients (stage I-III, n=34,616; stage IV, n =2,369) (Supplementary Figs. S1 & S2).

### Outcome Variable

The primary focus was trastuzumab receipt – recorded as a binary (Y/N) outcome variable. A patient who had a SACT record with a reference to receipt of trastuzumab (either alone or in combination with other drugs) was categorized as receiving trastuzumab (Y). Currently, SACT data lacks treatment indication detail, hence a timeframe restriction of 56 days prior to, and 1 year post diagnosis date was applied to increase confidence that trastuzumab use was for the primary invasive HER2+ breast cancer of interest. Supplementary Table S1 provides a breakdown of trastuzumab SACT data codes included for this outcome variable.

### Explanatory Variables

The main explanatory variable was deprivation category (proxy SES measure). In the NCRD, the IMD provides an area-based measure of relative deprivation for each small area (containing an average of 1,500 people) assigned based on postcode of residence at the time of cancer diagnosis.^39^ IMD is a widely-used composite index for classifying SES in England, based on characteristics of small areas.^40^ Whilst IMD is derived from seven domains (income, employment, education, skills and training, health deprivation and disability, crime, barriers to housing & services, and living environment), the NCRD only makes available the income domain (proportion of the population experiencing deprivation relating to low income – taken as both those out of work as well as those in work but who have low earnings).^41^ IMD income domain (henceforth referred to as IMD) was grouped into quintiles (1, least deprived; 5, most deprived). As IMD is updated periodically, the status closest to the date of breast cancer diagnosis was applied (i.e. IMD 2010 for diagnosis in 2012 and IMD 2015 for those diagnosed 2013-2017). Additional socio-demographic variables of interest were: age at diagnosis, ethnic group, rural/urban residence, and government region. Other potential covariates considered were: stage at diagnosis, tumor grade, presence of multiple tumors, number of comorbidities, ER status, whether women received surgery and/or chemotherapy within 6 months of diagnosis, whether each case was discussed at MDT, and year of diagnosis. Age was categorized into: <50, 50-59, 60-69, 70-79 and 80+ years old. Ethnicity was classified as: white, other ethnic group (Asian/British, Asian, Black/African/Caribbean/Black British, mixed/multiple ethic groups and other ethnic groups), and unknown (missing and unknown classifications). Rural/urban residence was defined as: rural village, hamlet, and isolated dwellings; rural town and fringe; urban city and town; and urban conurbation.^42^ The following nine government region were used: North West, North East, West Midlands, Yorkshire and the Humber, East Midlands, East of England, South East, South West, and London.^33^ TNM stage at diagnosis was categorized as: I, II, III, and IV. Grade was grouped as: well, moderately, and poorly differentiated, and other (undifferentiated, anaplastic, undetermined, or missing tumor grade). The multiple tumors variable took the value of 1 if the index breast cancer was the only cancer the individual had and was more than 1 if they had previously had (an)other cancer(s). ER status was classified as positive (at least one positive test, including borderline definitions), negative, or unknown. Number of comorbidities was determined from a weighted Charlson Comorbidity Index score,^43^ applied to conditions (with the exception of the index cancer) that resulted in hospital admissions in the period 78 to 6 months prior to diagnosis, and was categorized none, 1-2, and 3+. Receipt of surgery and chemotherapy within 6 months of cancer diagnosis was categorized as yes or no. Discussion at MDT was classified as yes, no, or missing. Finally, year of diagnosis explored temporal associations in treatment receipt.

### Statistical Analysis

Baseline demographic and clinical characteristics (number and percentage) were summarized for the full study cohort (stage I-IV). Descriptive statistics (number and percentage) are listed by all independent variables of interest for both the early (stage I-III) and metastatic (stage IV) sub-populations. Chi-square tests determined associations between socio-demographic/clinical characteristics and trastuzumab receipt in these two populations.

The likelihood of trastuzumab receipt in the (i) early-stage and; (ii) metastatic populations, by deprivation and all other socio-demographic/clinical characteristics was determined with univariable and multivariable logistic regression models. Any significant clinical and demographic variables in univariable analyses (likelihood ratio test (LRT) ≤0.05) were included in multivariate models. Deprivation, as the primary variable of interest, was forced into all models with IMD1 (least deprived) used as the reference group. Models report unadjusted and adjusted (multivariable) odds ratios (ORs; mvORs) with 95% CI(s) and p values. Model fit was checked, using Hosmer and Lemeshow χ^2^ tests, and variables contributing to poor fit were excluded. The Akaike Information Criterion (AIC) assisted decision making in instances where selection between competing models was needed. Variance inflation factors (VIF) were computed to provide an additional collinearity check; final model variables all had VIF<10. Throughout, a p value of ≤0.05 (two-sided tests) was considered statistically significant. In final multivariable models, a test for linear trend across deprivation categories was calculated.

Sensitivity analyses limited the stage I-III and stage IV cohorts to patients with: (i) date of incidence from April 2014 onwards to reflect the period when SACT reporting by hospital Trusts became mandatory (sensitivity analysis 1); and (ii) a refined (more definitive) HER2 status classification (i.e. positive only) to minimize the possibility of misclassification (sensitivity analysis 2).

All statistical analyses were conducted using STATA version 16.1 (StataCorp, College Station, Texas).

## Results

### Patient Characteristics

36,985 patients were diagnosed with stage I-IV HER2+ breast cancer between January 1^st^ 2012 and December 31^st^ 2017. Most women were of white ethnicity (88.1%); just under half were aged 50-69 (49.2%), a similar percentage resided in urban cities and towns (45.3%), and most had no comorbidities (82.3%). Much of the cohort had stage I-III tumors (93.6%); more than 90% graded as moderate or poorly differentiated (92.7%); and almost two-thirds as ER+ (63.7%). Population demographic and clinical characteristics are shown in Table 1.

### Trastuzumab Receipt: Early-Stage Population

Of the 34,616 women with early stage HER2+ disease diagnosed 2012-2017, 45.0% (n=15,567) received trastuzumab. Receipt increased over time from 34.1% of patients in 2012 to 44.2% in 2017. In univariate analyses trastuzumab receipt showed little patterning by SES. However, following adjustment for confounders (including clinical factors) in the multivariable model, a significant association between trastuzumab receipt and deprivation was seen (LRT=0.004). Patients’ resident in the most deprived areas were 10% less likely to receive trastuzumab than those resident in the least deprived areas (IMD 5 vs IMD 1; mvOR 0.90, 95% CI 0.83, 0.98) (Table 2). The test for linear trend across deprivation categories was significant (p = 0.002).

In sensitivity analyses, similar associations between trastuzumab receipt and deprivation were observed when restricting analyses to: (i) a HER2+ breast cancer diagnosis post mandatory SACT submission (April 2014) (sensitivity analysis 1) (IMD 5 vs IMD 1; mvOR 0.93, 95% CI 0.84, 1.03; LRT p=0.009), and (ii) were more defined when the refined HER2+ breast cancer definition (sensitivity analysis 2) was applied (IMD 5 vs IMD 1; mvOR 0.86, 95% CI 0.78, 0.95; LRT p=0.009). Both results were statistically significant (Supplementary Table S2).

Several other socio-demographic and clinical variables in the multivariable model also had statistically significant associations with reduced likelihood of trastuzumab receipt. These were: an older age (80+ vs <50 years old; mvOR 0.03, 95% CI 0.03, 0.03); three or more comorbidities (3+ vs 0 comorbidities; mvOR 0.38, 95% CI 0.32, 0.46) and; not being discussed at MDT (no vs yes; mvOR 0.81, 95% CI 0.75, 0.87). Ethnic group made a statistically significant contribution to the model. A negative ER status (negative vs positive; mvOR 2.29, 95% CI 2.16, 2.43), a higher stage cancer (stage III vs stage I; mvOR 2.57, 95% CI 2.38, 2.77) and not receiving surgery (no vs yes; mvOR 1.36, 95% CI 1.27, 1.45) were all associated with increased trastuzumab receipt. There was no patterning of trastuzumab receipt with urban/rural residence (Table 2).

### Trastuzumab Receipt: Metastatic Population

Of the 2,369 women with metastatic breast HER2+ breast cancer diagnosed 2012-2017, 44.8% (n=1,062) received trastuzumab. Receipt increased over time, from 29.1% of patients diagnosed in 2012 to 51.0% of patients diagnosed in 2017. In univariable and multivariable analyses, trastuzumab receipt did not vary significantly by deprivation quintile of residence (multivariable analysis, LRT p=0.225) even following adjustment for clinical factors, or have a significant linear trend (p=0.864). Odds of receipt followed a u-shaped pattern, being slightly below unity for deprivation quintiles 2-4, and reaching borderline significance for the middle category (IMD 3 vs IMD 1; mvOR 0.75, 95% CI 0.56, 1.00) (Table 3).

In sensitivity analyses, when restricting consideration to breast cancer diagnosis post mandatory SACT submission (April 2014) (sensitivity analysis 1), the univariable ORs were similar to the primary analysis, but the multivariable model did not have adequate fit (Supplementary Table S3). Restriction of the analysis to a refined HER2+ breast cancer definition (sensitivity analysis 2), showed no significant associations between IMD and trastuzumab receipt (Supplementary Table S3).

Several other demographic and clinical variables in the multivariable model had statistically significant associations with reduced likelihood of trastuzumab receipt in stage IV patients. These were: an older age (80+ vs <50 years old; mvOR 0.11, 95% CI 0.08, 0.16); a well differentiated tumor grade (well vs poorly differentiated grade; mvOR 0.33, 95% CI 0.14, 0.78); three or more comorbidities (3+ vs 0 comorbidities; mvOR 0.46, 95% CI 0.26, 0.80) and; no discussion at MDT (no vs yes; mvOR; 0.78, 95% CI 0.60, 1.00). Trastuzumab receipt was more common in women with ER negative tumors (negative vs positive; mvOR; 2.17, 95% CI 1.74, 2.69). Additionally, trastuzumab receipt was associated with both a white and non-white ethnicity (other ethnic group vs white ethnicity; mvOR; 1.00, 95% CI 0.71, 1.41) as well as residence in urban conurbations (urban conurbation vs urban city and town; mvOR 1.45, 95% CI 1.11, 1.87) and rural villages (rural village vs urban city and town; mvOR 1.48, 95% CI 1.06, 2.04) (Table 3).

## Discussion

This study addresses associations between IMD and other socio-demographic and clinical characteristics with trastuzumab receipt among a cohort of patients with HER2+ breast cancer in England during 2012-2017. It represents one of the few nationwide studies using SACT data and is the largest study internationally of trastuzumab receipt in a publicly funded healthcare system (and the second largest study on this topic after Du *et al*. (2011), which reported USA trastuzumab data up to 2005).^44^ This study found that women resident in areas of greater deprivation with early-stage HER2+ breast cancer were 10% less likely to receive trastuzumab compared to women resident in the least deprived areas. No clear or statistically significant associations between SES and trastuzumab receipt were found in the metastatic HER2+ cohort. A younger age was associated with increased trastuzumab receipt for all patients. Other associations of increased trastuzumab receipt were seen, in patients with both early and metastatic disease, who had: fewer comorbidities, an ER negative tumor, and who were discussed at MDT. For early-stage disease, a higher staged tumor and not receiving surgery within 6 months increased the likelihood of trastuzumab receipt. Whereas for metastatic disease, a moderate or poorly differentiated tumor grade and receiving surgery within 6 months was associated with increased trastuzumab receipt.

This population-based study significantly strengthens the evidence-base showing that socio-economic disparities of HER2+ breast cancer treatment occur even in healthcare systems where targeted therapy is free at the point of access. Our parallel study on socio-economic inequalities in non-small cell lung cancer (NSCLC) treatments in England over the same time period also found that greater deprivation was associated with reduced novel therapy use, though the magnitude of associations were starker.^45^ Given that socio-economic differences in treatment receipt may account for disparities in cancer survival and mortality,^46–48^ these inequities have important implications.

There is no clear, single explanation as to why trastuzumab receipt is less commonly used in women with early-stage breast cancer resident in more deprived areas or – as shown here – why receipt varies by age or rural-urban residence. Trastuzumab is well tolerated, toxicity is lower than cytotoxic chemotherapy therapy, and monitoring is in place to minimize cardiovascular complications.^49^ Theoretically, many women (regardless of socio-economic background) should tolerate trastuzumab. One barrier to receipt is HER2 testing access. However, as NICE recommends HER2 testing in England as routine,^50^ testing is well established in this cancer. It is possible that women residing in areas of high deprivation, or older women, maybe more likely to decline HER2 testing when offered. However, data from a previous systematic review and meta-analysis found only a small reduction in HER2 testing access by low SES – and this was not statistically significant.^17^

An alternative explanation for socio-economic (and, indeed, wider socio-demographic) patterning of trastuzumab receipt maybe that novel treatment analyses fail to consider the “fundamental causes” or “upstream” factors (i.e. unequal distribution of income and education) which generate inequity.^51^ Trastuzumab receipt is a “downstream intervention” focused on the final stages of the care pathway. The role of wider social determinants of health (SDoH) which also influence inequality generation and persistence are not addressed through trastuzumab licensing.^52^ Furthermore, MDTs may be an additional source of socio-economic biases (conscious or unconscious) in treatment decision-making.^53^ MDT discussion was important for increasing the likelihood of trastuzumab receipt, despite the fact that all accredited breast cancer units managed patients in England should be considered at MDT. This suggests that MDTs facilitate evidence-based, standardized clinical decision making around trastuzumab use.^54^ Previous work has discussed how MDT decision implementation can vary by deprivation status,^55^ however more work is needed to explore whether MDTs mitigate socio-economic biases in targeted treatment access. Finally, patient views of, and willingness to accept, trastuzumab maybe socio-economically/socio-demographically patterned. Treatment involves returning regularly to the hospital for up to a year; this may be challenging and/or less appealing to, the oldest patients or those with limited economic resources. Specific research on trastuzumab treatment decision-making is lacking, however a 2015 systematic review reported generally that convenience and transportation difficulties were key determinants of older adults’ decisions to accept or decline cancer treatment.^56^

Socio-economic associations in trastuzumab receipt varied by cancer stage, with an association evident for early stage, but not for metastatic disease. Potentially this reflects time since licensing. New drug interventions may become intervention generated inequality (IGI) examples when their introduction preferentially benefits those of higher SES with resources to gain priority access.^57^ IGIs are particularly concerning when treatments are new and wane over time as interventions become “standard practice” (Inverse Inequity Hypothesis).^58^ Minimal socio-economic disparities in metastatic compared to early-stage trastuzumab receipt may reflect first licencing in metastatic HER2+ disease in England in 2002 (access widened to early-stage breast cancer in 2006).^59,60^ This hypothesis cannot be considered nationally as SACT was only established in 2012.

This study is amongst the first to report English population-registry based data analysing receipt of a high-cost targeted treatment (trastuzumab) as well as exploring an emerging big data resource (SACT) – with a focus on socio-economic disparities. The national dataset coverage minimizes selection bias, improves data completeness, and enhances study validity. Despite these strengths, there are several limitations. First, early SACT data completeness prior to mandated Trust submission post April 2014 is uncertain and may explain apparent low overall trastuzumab receipt.^37^ However, sensitivity analyses for early-stage disease confirmed that associations with deprivation were not impacted by time. Second, NCRD data collection across healthcare providers can vary.^35^ This may explain why surgery rates in early-stage breast cancer were lower than anticipated. Third, it is possible that recording of trastuzumab by hospital Trusts is biased by deprivation category or other socio-demographic factors. However, given the comparability in demographic characteristics between patients both with and without SACT information recorded (not shown) and the fact that hospital catchment areas have diverse populations, this seems unlikely. Fourth, IMD was at area, rather than patient-level and only considered a single domain of deprivation (income), so care is needed to avoid the ecological fallacy and the assumption that similar associations would be observed with other SES measures, especially those at the individual level (e.g. education level, employment status).^61^ Moreover, ethnicity was based on information recorded in hospital records; whilst quality of ethnicity data for the period of the study is considered better than in earlier years,^62^ 4.2% of patients were recorded as “unknown” ethnic group (and only 7.7% as non-white ethnic group), which may have introduced misclassification. Combining of non-white ethnicities into one group for the purpose of analysis meant that variation between the constituent ethnic groups could not be investigated; this in turn limits the ability to target interventions tackling inequalities based on ethnicity. Fifth, it was not possible to account for all factors serving as a barrier to treatment receipt (e.g. a low performance status). Sixth, whilst comorbidity presence was adjusted for in models, the Charlson Comorbidity Index (computed from hospital admissions in the period 78 to 6 months prior to cancer diagnosis) is a crude measure, so there is likely residual confounding by fitness for treatment.^63^ Finally, this study reports a snapshot of trastuzumab receipt pre-COVID-19 pandemic in one country. Results may not be generalizable to other novel high-cost targeted treatments, countries, or the period since 2017.

Future research has several priorities: (i) exploring whether inequities in trastuzumab receipt explain observed disparities in outcomes (survival and quality of life); (ii) seeking to better understand “causal mechanisms” underpinning current findings; and (iii) extending inequity evaluations to other novel therapies (including those where predictive biomarker testing is not undertaken) and other cancers. From a policy and practice perspective, an increasing focus on implementing effective strategies and policies to overcome unfair novel treatment access is needed. This is pertinent given that targeted treatments are expanding for HER2 and other breast cancer subtypes (e.g. abemaciclib). Timely monitoring of novel treatment receipt to ensure that inequities do not become established is needed. Solutions likely require attention from patient, NHS provider, healthcare system, and wider society levels. Application of approaches like Intervention Mapping (a framework that uses theory and evidence to support intervention development) would be of value to inform the systematic development and testing of solutions. As an initial step, improved understanding of the determinants of utilization is required.^64^ Later stages could consider adaption of interventions successfully applied in other contexts^65,66^ to improve medication utilization. Examples include e.g. targeted health literacy interventions to improve patient participation in shared treatment decision making, increased education of clinicians in the SDoH, and use of patient navigators to support more disadvantaged patients. Finally, interventions which may improve ease of trastuzumab use (e.g. shorter duration of therapy)^67^ could also be of value in reducing potential socio-economic barriers (e.g. financial).

## Conclusions

There are socio-demographic disparities HER2+ breast cancer trastuzumab receipt in England. In both early and metastatic disease, older women, with more comorbidities, ER positive disease and who are not discussed at MDT are less likely to receive trastuzumab. Reduced trastuzumab receipt amongst those residents in more deprived areas was also observed in women with early-stage disease. These inequities are present, despite biomarker driven guidelines and trastuzumab being free at the point of delivery in the publicly funded NHS. National policies to address inequalities in trastuzumab and other novel breast cancer treatments are urgently needed. Policies should focus on ensuring fair access regardless of place of residence, socio-demographic characteristics and/or cancer stage.

## Supplementary Material

Supplementary Fig. S1

Supplementary Fig. S2

Supplementary Materials and Methods

Supplementary Table S1

Supplementary Table S2

Supplementary Table S3

## Figures and Tables

**Table 1:** Demographic and clinical characteristics of women with stage I-IV HER2+ breast cancer diagnosed between 01/01/2012 - 31/12/2017 (n = 36,985)

	Stage I-IV 36,985 (100.00)	Stage I-III 34,616 (93.59)	Stage IV 2,369 (6.41)
Characteristic		Number (%)	
**Deprivation^a^**			
1 (Least Deprived)	8,454 (22.86)	7,970 (23.02)	484 (20.43)
2	8,267 (22.35)	7,786 (22.49)	481 (20.30)
3	7,469 (20.19)	7,024 (20.29)	445 (18.78)
4	6,814 (18.42)	6,299 (18.20)	515 (21.74)
5 (Most Deprived)	5,981 (16.17)	5,537 (16.00)	444 (18.74)
**Age at Diagnosis (Years)**			
<50	8,962 (24.23)	8,466 (24.46)	496 (20.94)
50 − 59	9,132 (24.69)	8,617 (24.89)	515 (21.74)
60 − 69	9,058 (24.49)	8,592 (24.82)	466 (19.67)
70 − 79	5,997 (16.21)	5,489 (15.86)	508 (21.44)
80+	3,836 (10.37)	3,452 (9.97)	384 (16.21)
**Ethnicity**			
White	32,565 (88.05)	30,500 (88.11)	2,065 (87.17)
Other Ethnic Group^b^	2,852 (7.71)	2,653 (7.66)	199 (8.40)
Unknown^c^	1,568 (4.24)	1,463 (4.23)	105 (4.43)
**Rural/Urban Residence**			
Rural Village, Hamlet & Isolated Dwellings	4,008 (10.84)	3,772 (10.90)	236 (9.96)
Rural Town & Fringe	3,942 (10.66)	3,686 (10.65)	256 (10.81)
Urban City & Town	16,744 (45.27)	15,666 (45.26)	1,078 (45.50)
Urban Conurbation	12,291 (33.23)	11,492 (33.20)	799 (33.73)
**Government Region**			
North West	5,937 (16.05)	5,583 (16.13)	354 (14.94)
North East	2,437 (6.59)	2,287 (6.61)	150 (6.33)
West Midlands	3,929 (10.62)	3,683 (10.64)	246 (10.38)
Yorkshire & the Humber	3,310 (8.95)	3,056 (8.83)	254 (10.72)
East Midlands	3,128 (8.46)	2,958 (8.55)	170 (7.18)
East of England	5,010 (13.55)	4,666 (13.48)	344 (14.52)
South East	5,711 (15.44)	5,316 (15.36)	395 (16.67)
South West	4,049 (10.95)	3,836 (11.08)	213 (8.99)
London	3,474 (9.39)	3,231 (9.33)	243 (10.26)
**Stage at Diagnosis**			
I	13,094 (35.40)	13,094 (37.83)	----------
II	16,663 (45.05)	16,663 (48.14)	----------
III	4,859 (13.14)	4,859 (14.04)	----------
IV	2,369 (6.41)	----------	2,369 (100.00)
**Grade**			
Well Differentiated	2,034 (5.50)	1,999 (5.77)	35 (1.48)
Moderately Differentiated	16,077 (43.47)	15,122 (43.69)	955 (40.31)
Poorly Differentiated	18,217 (49.26)	16,989 (49.08)	1,228 (51.84)
Other^d^	657 (1.78)	506 (1.46)	151 (6.37)
**Multiple Tumors^e^**			
1 Tumor Only	32,570 (88.06)	30,533 (88.20)	2,037 (85.99)
>1 Tumors	4,415 (11.94)	4,083 (11.80)	332 (14.01)
**ER Status**			
Positive^f^	23,557 (63.69)	22,299 (64.42)	1,258 (53.10)
Negative	7,912 (21.39)	7,233 (20.89)	679 (28.66)
Unknown^g^	5,516 (14.91)	5,084 (14.69)	432 (18.24)
**No. of Comorbidities (between 78 to 6** **months prior to diagnosis)^h^**			
None	30,423 (82.26)	28,494 (82.31)	1,929 (81.43)
1 − 2	5,369 (14.52)	5,016 (14.49)	353 (14.90)
3+	1,193 (3.23)	1,106 (3.20)	87 (3.67)
**Discussed at MDT**			
Yes	25,997 (70.29)	24,672 (71.27)	1,325 (55.93)
No	5,789 (15.65)	5,352 (15.46)	437 (18.45)
Missing	5,199 (14.06)	4,592 (13.27)	607 (25.62)
**Diagnosis Year**			
2012	4,099 (11.08)	3,810 (11.01)	289 (12.20)
2013	4,982 (13.47)	4,638 (13.40)	344 (14.52)
2014	5,705 (15.43)	5,325 (15.38)	380 (16.04)
2015	6,561 (17.74)	6,144 (17.75)	417 (17.60)
2016	7,476 (20.21)	7,031 (20.31)	445 (18.78)
2017	8,162 (22.07)	7,668 (22.15)	494 (20.85)
**Treatment^i^**			
Received Chemotherapy^j,k^	22,893 (61.90)	21,353 (61.69)	1,540 (65.01)
Received Surgery^j,k^	28,738 (77.70)	28,294 (81.74)	444 (18.74)
Received Trastuzumab^l^	16,629 (44.96)	15,567 (44.97)	1,062 (44.83)

a Refers to IMD (income domain). For diagnosis year 2012, IMD_2010 was used and for diagnosis years 2013-2017, IMD_2015 was used.

b Other ethnic group refers to Asian/British Asian, Black/African/Caribbean/Black British, mixed/multiple ethnic groups and other ethnic groups.

c Unknown ethnicity refers to unknown and missing ethnicity classifications.

d Other grade refers to undifferentiated, anaplastic, undetermined, and missing tumor grades.

e Refers to the number of tumors other than the index breast cancer (1 tumor only).

f Refers to at least one positive ER test and includes borderline definitions.

g Refers to unknown, not performed, or missing ER status.

h Refers to Charlson Comorbidity Index.

i Not exclusive, so do not total analytical cohort totals.

j Within six months of diagnosis. Surgery receipt beyond 6 months (e.g. following neo-adjuvant chemotherapy) not captured.

k Data from NCRD.

l Data from SACT.

Abbreviations: IMD: Index of Multiple Deprivation (income domain); HER2: Human epidermal growth factor receptor 2; NCRD: National Cancer Registry Database; SACT: Systemic Anti-Cancer Therapy Database.

**Table 2:** Early-stage Disease: Likelihood (OR with 95% CI and p values from logistic regression) of receiving trastuzumab by deprivation and adjusted for: age, ethnicity, rural/urban residence, government region, stage at diagnosis, whether received surgery, ER status, comorbidities, whether discussed at MDT, and diagnosis year for women with stage I-III HER2+ breast cancer who were diagnosed between 01/01/2012 - 31/12/2017 (n = 34,616)

				Unadjusted			Adjusted		
	Number (%) receiving Trastuzumab n = 15,567 (44.97)	Number (%) not receiving Trastuzumab n = 19,049 (55.03)	P Value^a^	OR	95% CI	P Value^b^	OR	95% CI	P Value^b^
**Deprivation^c^**			0.021			**0.020**			**0.004**
1 (Least Deprived)	3,605 (45.23)	4,365 (54.77)		1.00	----- -----	-----	1.00	----- -----	-----
2	3,525 (45.27)	4,261 (54.73)		1.00	0.94 – 1.07	0.958	1.00	0.94 – 1.08	0.910
3	3,039 (43.27)	3,985 (56.73)		0.92	0.87 – 0.99	0.016	0.90	0.84 – 0.96	0.003
4	2,847 (45.20)	3,452 (54.80)		1.00	0.93 – 1.07	0.967	0.93	0.86 – 1.00	0.050
5 (Most Deprived)	2,551 (46.07)	2,986 (53.93)		1.03	0.97 – 1.11	0.335	0.90	0.83 – 0.98	0.016
**Age at Diagnosis (Years)**			<0.001			**<0.001**			**<0.001**
<50	5,167 (61.03)	3,299 (38.97)		1.00	----- -----	-----	1.00	----- -----	-----
50-59	4,625 (53.67)	3,992 (46.33)		0.74	0.70 – 0.79	<0.001	0.79	0.74 – 0.84	<0.001
60-69	3,912 (45.53)	4,680 (54.47)		0.53	0.50 – 0.58	<0.001	0.59	0.55 – 0.63	<0.001
70-79	1,676 (30.53)	3,813 (69.47)		0.28	0.26 – 0.30	<0.001	0.27	0.25 – 0.29	<0.001
80+	187 (5.42)	3,265 (94.58)		0.04	0.03 – 0.04	<0.001	0.03	0.03 – 0.03	<0.001
**Ethnicity**			<0.001			**<0.001**			**<0.001**
White	13,720 (44.98)	16,780 (55.02)		1.00	----- -----	-----	1.00	----- -----	-----
Other Ethnic Group^d^	1,393 (52.51)	1,260 (47.49)		1.35	1.25 – 1.46	<0.001	0.93	0.84 – 1.02	0.110
Unknown^e^	454 (31.03)	1,009 (68.97)		0.55	0.49 – 0.62	<0.001	0.47	0.41 – 0.53	<0.001
**Rural/Urban Residence**			<0.001			**<0.001**			**0.000**
Rural Village, Hamlet & Isolated Dwellings	1,688 (44.75)	2,084 (55.25)		1.05	0.97 – 1.12	0.218	1.03	0.95 – 1.12	0.466
Rural Town & Fringe	1,627 (44.14)	2,059 (55.86)		1.02	0.95 – 1.10	0.584	1.07	0.98 – 1.16	0.121
Urban City & Town	6,837 (43.64)	8,829 (56.36)		1.00	----- -----	-----	1.00	----- -----	-----
Urban Conurbation	5,415 (47.12)	6,077 (52.88)		1.15	1.10 – 1.21	<0.001	1.16	1.09 – 1.25	<0.001
**Government ** **Region**			<0.001			**<0.001**			**<0.001**
North West	2,509 (44.94)	3,074 (55.06)		1.00	----- -----	-----	1.00	----- -----	-----
North East	897 (39.22)	1,390 (60.78)		0.79	0.72 – 0.87	<0.001	0.70	0.63 – 0.79	<0.001
West Midlands	1,543 (41.90)	2,140 (58.10)		0.88	0.81 – 0.96	0.004	0.83	0.75 – 0.91	<0.001
Yorkshire & the Humber	1,687 (55.20)	1,369 (44.80)		1.51	1.38 – 1.65	<0.001	1.41	1.28 – 1.56	<0.001
East Midlands	1,395 (47.16)	1,563 (52.84)		1.09	1.00 – 1.20	0.050	1.09	0.99 – 1.21	0.090
East of England	1,983 (42.50)	2,683 (57.50)		0.91	0.84 – 0.98	0.013	0.88	0.80 – 0.97	0.008
South East	2,346 (44.13)	2,970 (55.87)		0.97	0.90 – 1.04	0.396	1.00	0.92 – 1.10	0.945
South West	1,685 (43.93)	2,151 (56.07)		0.96	0.88 – 1.04	0.331	1.07	0.97 – 1.18	0.186
London	1,522 (47.11)	1,709 (52.89)		1.09	1.00 – 1.19	0.049	0.92	0.83 – 1.03	0.139
**Stage at Diagnosis**			<0.001			**<0.001**			**<0.001**
I	4,680 (35.74)	8,414 (64.26)		1.00	----- -----	-----	1.00	----- -----	-----
II	8,189 (49.14)	8,474 (50.86)		1.74	1.66 – 1.82	<0.001	1.91	1.81 – 2.01	<0.001
III	2,698 (55.53)	2,161 (44.47)		2.24	2.10 – 2.40	<0.001	2.57	2.38 2.77	<0.001
**Grade**			<0.001			**<0.001**			-----^l^
Well Differentiated	232 (11.61)	1,767 (88.39)		0.10	0.09 – 0.12	<0.001	-----	----- -----	-----
Moderately Differentiated	5,575 (36.87)	9,547 (63.13)		0.46	0.44 – 0.48	<0.001	-----	----- -----	-----
Poorly Differentiated	9,533 (56.11)	7,456 (43.89)		1.00	----- -----	-----	-----	----- -----	-----
Other^f^	227 (44.86)	279 (55.14)		0.64	0.53 – 0.76	<0.001	-----	----- -----	-----
**Received Surgery within 6 Months of Diagnosis^g^**			<0.001			**<0.001**			**<0.001**
Yes	12,536 (44.31)	15,758 (55.69)		1.00	----- -----	-----	1.00	----- -----	-----
No	3,031 (47.94)	3,291 (52.06)		1.16	1.10 – 1.22	<0.001	1.36	1.27 – 1.45	<0.001
**Multiple Tumors^h^**			<0.001			**<0.001**			-------^l^
1 Tumor Only	14,135 (46.29)	16,398 (53.71)		1.00	----- -----	-------	-------	-------	-------
>1 Tumors	1,432 (35.07)	2,651 (64.93)		0.63	0.58 – 0.67	<0.001	-------	----- -----	-------
**ER Status**			<0.001			**<0.001**			**<0.001**
Positive^i^	8,859 (39.73)	13,440 (60.27)		1.00	----- -----	-----	1.00	----- -----	-----
Negative	4,204 (58.12)	3,029 (41.88)		2.11	2.00 – 2.22	<0.001	2.29	2.16 – 2.43	<0.001
Unknown^j^	2,504 (49.25)	2,580 (50.75)		1.47	1.39 – 1.57	<0.001	1.42	1.33 – 1.53	<0.001
**No. of Comorbidities (Between 78 to 6 Months Prior to Diagnosis)^k^**			<0.001			**<0.001**			**<0.001**
0	13,614 (47.78)	14,880 (52.22)		1.00	------ -----	-----	1.00	----- -----	-----
1-2	1,776 (35.41)	3,240 (64.59)		0.60	0.56 – 0.64	<0.001	0.83	0.77 – 0.89	<0.001
3+	177 (16.00)	929 (84.00)		0.21	0.18 – 0.24	<0.001	0.38	0.32 – 0.46	<0.001
**Discussed at MDT**			<0.001			**<0.001**			**<0.001**
Yes	11,584 (46.95)	13,088 (53.05)		1.00	----- -----	-----	1.00	----- -----	-----
No	2,222 (41.52)	3,130 (58.48)		0.80	0.76 – 0.85	<0.001	0.81	0.75 – 0.87	<0.001
Missing	1,761 (38.35)	2,831 (61.65)		0.70	0.66 – 0.75	<0.001	0.56	0.52 – 0.60	<0.001
**Diagnosis Year**			<0.001			**<0.001**			**<0.001**
2012	1,299 (34.09)	2,511 (65.91)		0.65	0.60 – 0.71	<0.001	0.50	0.46 – 0.55	<0.001
2013	2,155 (46.46)	2,483 (53.54)		1.10	1.02 – 1.18	0.013	0.94	0.86 – 1.02	0.128
2014	2,521 (47.34)	2,804 (52.66)		1.14	1.06 – 1.22	<0.001	1.04	0.96 – 1.13	0.300
2015	2,892 (47.07)	3,252 (52.93)		1.12	1.05 – 1.20	0.001	1.10	1.02 – 1.19	0.012
2016	3,314 (47.13)	3,717 (52.87)		1.13	1.06 – 1.20	<0.001	1.15	1.07 – 1.23	<0.001
2017	3,386 (44.16)	4,282 (55.84)		1.00	----- -----	-----	1.00	----- -----	-----

a Chi-square P value.

b Bolded P values are from LRT of the variable’s contribution to the model. Unbolded P values are from a test of whether the OR is different from 1.

c Refers to IMD (income domain). For diagnosis year 2012, IMD_2010 was used and for diagnosis years 2013-2017, IMD_2015 was used.

d Other ethnic group refers to Asian/British Asian, Black/African/Caribbean/Black British, mixed/multiple ethnic groups, and other ethnic groups.

e Unknown ethnicity refers to missing and unknown ethnicity classifications.

f Other grade refers to undifferentiated, anaplastic, undetermined, and missing tumor grades.

g Surgery receipt beyond 6 months (e.g. following neo-adjuvant chemotherapy) not captured.

h Refers to the number of tumors other than the index breast cancer (1 tumor only).

i Refers to at least one positive ER test and includes borderline definitions.

j Refers to unknown, not performed, or missing ER status.

k Defined using the Charlson Comorbidity Index.

l Variable not included in the adjusted analysis as resulted in poor model fit (assessed using Hosmer and Lemeshow χ^2^ test).

Abbreviations: ER: Estrogen receptor; HER2: Human epidermal growth factor receptor 2; IMD: Index of multiple deprivation (income domain); LRT: Likelihood ratio test; n: Number; MDT: Multidisciplinary team; OR: Odds ratio; CI: Confidence interval.

**Table 3:** Metastatic Disease: Likelihood (OR with 95% CI and p values from logistic regression) of receiving trastuzumab by deprivation and adjusted for: age, ethnicity, rural/urban residence, government region, grade, whether received surgery, ER status, comorbidities, whether discussed at MDT, and diagnosis year for women with stage IV HER2+ breast cancer diagnosed between 01/01/2012 - 31/12/2017 (n = 2,369)

				Unadjusted			Adjusted		
	Number (%) receiving Trastuzumab n = 1,062 (44.83)	Number (%) not receiving Trastuzumab n = 1,307 (55.17)	P Value^a^	OR	95% CI	P Value^b^	OR	95% CI	P Value^b^
**Deprivation^c^**			0.314			**0.312**			**0.225**
1 (Least Deprived)	224 (46.28)	260 (53.72)		1.00	----- -----	-----	1.00	----- -----	-----
2	215 (44.70)	266 (55.30)		0.94	0.73 – 1.21	0.622	0.85	0.64 – 1.13	0.270
3	182 (40.90)	263 (59.10)		0.80	0.62 – 1.04	0.099	0.75	0.56 – 1.00	0.052
4	229 (44.47)	286 (55.53)		0.93	0.72 – 1.19	0.565	0.84	0.63 – 1.12	0.232
5 (Most Deprived)	212 (47.75)	232 (52.25)		1.06	0.82 – 1.37	0.655	1.00	0.74 – 1.37	0.977
**Age at Diagnosis (Years)**			<0.001			**<0.001**			**<0.001**
<50	314 (63.31)	182 (36.69)		1.00	----- -----	-----	1.00	----- -----	-----
50–59	285 (55.34)	230 (44.66)		0.72	0.56 – 0.92	0.010	0.67	0.51 – 0.87	0.003
60–69	223 (47.85)	243 (52.15)		0.53	0.41 – 0.69	<0.001	0.55	0.41 – 0.73	<0.001
70–79	171 (33.66)	337 (66.34)		0.29	0.23 – 0.38	<0.001	0.27	0.20 – 0.36	<0.001
80+	69 (17.97)	315 (82.03)		0.13	0.09 – 0.17	<0.001	0.11	0.08 – 0.16	<0.001
**Ethnicity**			<0.001			**<0.001**			**0.002**
White	923 (44.70)	1,142 (55.30)		1.00	----- -----	-----	1.00	----- -----	-----
Other Ethnic Group^d^	109 (54.77)	90 (45.23)		1.50	1.12 – 2.01	0.007	1.00	0.71 – 1.41	0.998
Unknown^e^	30 (28.57)	75 (71.43)		0.49	0.32 – 0.76	0.001	0.44	0.27 – 0.71	0.001
**Rural/Urban Residence**			0.042			**0.042**			**0.010**
Rural Village, Hamlet & Isolated Dwellings	116 (49.15)	120 (50.85)		1.31	0.99 – 1.74	0.058	1.48	1.06 – 2.04	0.020
Rural Town & Fringe	107 (41.80)	149 (58.20)		0.98	0.74 – 1.29	0.862	1.07	0.79 – 1.46	0.665
Urban City & Town	457 (42.39)	621 (57.61)		1.00	----- -----	-----	1.00	----- -----	-----
Urban Conurbation	382 (47.81)	417 (52.19)		1.24	1.04 – 1.50	0.020	1.45	1.11 – 1.87	0.005
**Government ** **Region**			0.003			**0.002**			**<0.001**
North West	170 (48.02)	184 (51.98)		1.00	----- -----	-----	1.00	----- -----	-----
North East	63 (42.00)	87 (58.00)		0.78	0.53 – 1.15	0.215	0.60	0.39 – 0.93	0.023
West Midlands	85 (34.55)	161 (65.45)		0.57	0.41 – 0.80	0.001	0.38	0.26 – 0.56	<0.001
Yorkshire & the Humber	130 (51.18)	124 (48.82)		1.13	0.82 – 1.57	0.442	0.84	0.58 – 1.21	0.350
East Midlands	89 (52.35)	81 (47.65)		1.19	0.82 – 1.72	0.354	0.95	0.62 – 1.46	0.815
East of England	143 (41.57)	201 (58.43)		0.77	0.57 – 1.04	0.087	0.68	0.48 – 0.99	0.042
South East	185 (46.84)	210 (53.16)		0.95	0.72 – 1.27	0.745	0.81	0.57 – 1.15	0.235
South West	88 (41.31)	125 (58.69)		0.76	0.54 – 1.07	0.121	0.84	0.56 – 1.27	0.418
London	109 (44.86)	134 (55.14)		0.88	0.63 – 1.22	0.446	0.56	0.38 – 0.83	0.004
**Grade**			<0.001			**<0.001**			**0.030**
Well Differentiated	8 (22.86)	27 (77.14)		0.30	0.14 – 0.67	0.003	0.33	0.14 – 0.78	0.011
Moderately Differentiated	386 (40.42)	569 (59.58)		0.69	0.58 – 0.82	0.000	0.86	0.71 – 1.04	0.117
Poorly Differentiated	609 (49.59)	619 (50.41)		1.00	----- -----	-----	1.00	----- -----	-----
Other^f^’	59 (39.07)	92 (60.93)		0.65	0.46 – 0.92	0.015	0.87	0.59 – 1.29	0.491
**Received Surgery within 6 Months of**			<0.001			**0.000**			**0.016**
**Diagnosis** **^g^**									
Yes	234 (52.70)	210 (47.30)		1.48	1.20 – 1.82	<0.001	1.33	1.05 – 1.69	0.016
No	828 (43.01)	1,097 (56.99)		1.00	----- -----	-----	1.00	----- -----	-----
**Multiple Tumors^h^**			0.001			**0.001**			-----^l^
1 Tumor Only	941 (46.20)	1,096 (53.80)		1.00	----- -----	-----	-----	----- -----	-----
>1 Tumors	121 (36.45)	211 (63.55)		0.67	0.53 – 0.85	0.001	-----	----- -----	-----
**ER Status**			<0.001			**<0.001**			**<0.001**
Positive^i^	482 (38.31)	776 (61.69)		1.00	----- -----	-----	1.00	----- -----	-----
Negative	367 (54.05)	312 (45.95)		1.89	1.57 – 2.29	<0.001	2.17	1.74 – 2.69	<0.001
Unknown^j^	213 (49.31)	219 (50.69)		1.57	1.26 – 1.95	<0.001	1.49	1.16 – 1.93	0.002
**No. of Comorbidities (Between 78 to 6** **Months Prior to Diagnosis)k**			<0.001			**<0.001**			**0.015**
0	906 (46.97)	1,023 (53.03)		1.00	----- -----	-----	1.00	----- -----	-----
1-2	136 (38.53)	217 (61.47)		0.71	0.56 – 0.89	0.004	0.94	0.72 – 1.22	0.634
3+	20 (22.99)	67 (77.01)		0.34	0.20 – 0.56	<0.001	0.46	0.26 – 0.80	0.006
**Discussed at MDT**			0.001			**0.001**			**<0.001**
Yes	637 (48.08)	688 (51.92)		1.00	----- -----	-----	1.00	----- -----	-----
No	188 (43.02)	249 (56.98)		0.82	0.66 – 1.01	0.067	0.78	0.60 – 1.00	0.053
Missing	237 (39.04)	370 (60.96)		0.69	0.57 – 0.84	<0.001	0.57	0.46 – 0.72	<0.001
**Diagnosis Year**			<0.001			**<0.001**			**<0.001**
2012	84 (29.07)	205 (70.93)		0.39	0.29 – 0.54	<0.001	0.26	0.19 – 0.37	<0.001
2013	143 (41.57)	201 (58.43)		0.68	0.52 – 0.90	0.007	0.47	0.34 – 0.65	<0.001
2014	163 (42.89)	217 (57.11)		0.72	0.55 – 0.94	0.017	0.52	0.38 – 0.70	<0.001
2015	204 (48.92)	213 (51.08)		0.92	0.71 – 1.19	0.529	0.76	0.56 – 1.02	0.066
2016	216 (48.54)	229 (51.46)		0.91	0.70 – 1.17	0.449	0.82	0.61 – 1.09	0.163
2017	252 (51.01)	242 (48.99)		1.00	----- -----	-----	1.00	----- -----	-----

a Chi-square P value.

b Bolded P values are from LRT of the variable’s contribution to the model. Unbolded P values are from a test of whether the OR is different from 1.

c Refers to IMD (income domain). For diagnosis year 2012, IMD_2010 was used and for diagnosis years 2013-2017, IMD_2015 was used.

d Other ethnic group refers to Asian/British Asian, Black/African/Caribbean/Black British, mixed/multiple ethnic groups, and other ethnic groups.

e Unknown ethnicity refers to missing and unknown ethnicity classifications.

f Other grade refers to undifferentiated, anaplastic, undetermined, and missing tumor grades.

g Surgery receipt beyond 6 months (e.g. following neo-adjuvant chemotherapy) not captured.

h Refers to the number of tumors other than the index breast cancer (1 tumor only).

i Refers to at least one positive ER test and includes borderline definitions.

j Refers to unknown, not performed, or missing ER status.

k Defined using the Charlson Comorbidity Index.

l Variable not included in the adjusted analysis as resulted in poor model fit (assessed using Hosmer and Lemeshow χ^2^ test).

Abbreviations: ER: Estrogen receptor; HER2: Human epidermal growth factor receptor 2; IMD: Index of multiple deprivation (income domain); LRT: Likelihood ratio test; n: Number; MDT: Multidisciplinary team; OR: Odds ratio; CI: Confidence interval.

## Data Availability

The data analyzed in this study are available from the current Data Controller, NHS England. Restrictions apply to the availability of these data, which were used under the license for this study only; the authors are not permitted to share these data.

## References

[1] Crimini E, Repetto M, Aftimos P, Botticelli A, Marchetti P, Curigliano G (2021). Precision medicine in breast cancer: from clinical trials to clinical practice. Cancer Treat Rev.

[2] Arteaga CL, Sliwkowski MX, Osborne CK, Perez EA, Puglisi F, Gianni L (2011). Treatment of HER2 positive breast cancer: current status and future perspectives. Nat Rev Clin Oncol.

[3] Hudis CA (2007). Trastuzumab-mechanism of action and use in clinical practice. N Engl J Med.

[4] Slamon D, Pegram M (2001). Rationale for trastuzumab (Herceptin) in adjuvant breast cancer trials. Semin Oncol.

[5] Slamon DJ, Clark GM, Wong SG, Levin WJ, Ullrich A, McGuire WL (1987). Human breast cancer: correlation of relapse and survival with amplification of the HER-2/neu oncogene. Science.

[6] Cardoso F, Kyriakides S, Ohno S, Penault-Llorca F, Poortmans P, Rubio IT (2019). Early breast cancer: ESMO clinical practice guidelines for diagnosis, treatment and follow-up. Ann Oncol.

[7] Gennari A, André F, Barrios CH, Cortés J, de Azambuja E, DeMichele A (2021). ESMO clinical practice guideline for the diagnosis, staging and treatment of patients with metastatic breast cancer. Ann Oncol.

[8] Bradley R, Braybrooke J, Gray R, Hills R, Liu Z, Peto R (2021). Trastuzumab for early-stage, HER2-positive breast cancer: a meta-analysis of 13,864 women in seven randomised trials. Lancet Oncol.

[9] Balduzzi S, Mantarro S, Guarneri V, Tagliabue L, Pistotti V, Moja L (2014). Trastuzumab-containing regimens for metastatic breast cancer. Cochrane Database Syst Rev.

[10] Van Loon AJ, Goldbohm RA, Van den Brandt PA (1994). Socioeconomic status and breast cancer incidence: a prospective cohort study. Int J Epidemiol.

[11] Lagerlund M, Bellocco R, Karlsson P, Tejler G, Lambe M (2005). Socio-economic factors and breast cancer survival-a population-based cohort study (Sweden). Cancer Causes Control.

[12] Dreyer MS, Nattinger AB, McGinley EL, Pezzin LE (2018). Socioeconomic status and breast cancer treatment. Breast Cancer Res Treat.

[13] Neuner JM, Kong A, Blaes A, Riley D, Chrischilles E, Smallwood A (2019). The association of socioeconomic status with receipt of neoadjuvant chemotherapy. Breast Cancer Res Treat.

[14] Singh S, Sridhar P (2021). A narrative review of sociodemographic risk and disparities in screening, diagnosis, treatment, and outcomes of the most common extrathoracic malignancies in the United States. J Thorac Dis.

[15] All Party Parliamentary Group on Breast Cancer A mixed picture: an inquiry into geographical inequalities and breast cancer.

[16] Tuckson RV, Newcomer L, de Sa JM (2013). Accessing genomic medicine: affordability, diffusion, and disparities. JAMA.

[17] Norris RP, Dew R, Sharp L, Greystoke A, Rice S, Johnell K (2020). Are there socio-economic inequalities in utilization of predictive biomarker tests and biological and precision therapies for cancer? A systematic review and meta-analysis. BMC Med.

[18] Kaufman PA, Hurvitz SA, O’Shaughnessy J, Mason G, Yardley DA, Brufsky AM (2021). Baseline characteristics and first-line treatment patterns in patients with HER2-positive metastatic breast cancer in the SystHERs registry. Breast Cancer Res Treat.

[19] Nguy S, Wu SP, Oh C, Gerber NK (2021). Outcomes of HER2-positive non-metastatic breast cancer patients treated with anti-HER2 therapy without chemotherapy. Breast Cancer Res Treat.

[20] Martin AP, Downing J, Cochrane M, Collins B, Francis B, Haycox A (2018). Trastuzumab uptake in HER2-positive breast cancer patients: a systematic review and meta-analysis of observational studies. Crit Rev Oncol Hematol.

[21] Diao Y, Lin M, Xu K, Huang J, Wu X, Li M (2021). How government health insurance coverage of novel anti-cancer medicines benefited patients in China - a retrospective analysis of hospital clinical data. BMC Health Serv Res.

[22] Shang L, Lin Y, Fang W, Liu Y, Bao Y, Li X (2023). How national health insurance coverage policy affected the use of trastuzumab and rituximab in China: a bicentric retrospective study. Risk Manag Health Policy.

[23] Singh P, Pasricha R, Pandey L, Joseph D, Ibrahim A, Bhadoria AS (2021). Socioeconomic factors affecting trastuzumab usage in patients with breast cancer in a resource constrained setting in north India. Int J Community Med Public Health.

[24] Kumachev A, Trudeau ME, Chan KKW (2016). Associations among socioeconomic status, patterns of care and outcomes in breast cancer patients in a universal health care system: Ontario’s experience. Cancer.

[25] Goldhar HA, Yan AT, Ko DT, Earle CC, Tomlinson GA, Trudeau ME (2016). The temporal risk of heart failure associated with adjuvant trastuzumab in breast cancer patients: a population study. JNCI.

[26] Thavendiranathan P, Abdel-Qadir H, Fischer HD, Camacho X, Amir E, Austin PC (2016). Breast cancer therapy-related cardiac dysfunction in adult women treated in routine clinical practice: a population-based cohort study. J Clin Oncol.

[27] Lu CY, Srasuebkul P, Drew AK, Chen K, Ward RL, Pearson SA (2013). Trastuzumab therapy in Australia: which patients with HER2+ metastatic breast cancer are assessed for cardiac function?. Breast.

[28] Tang M, Schaffer AL, Kiely BE, Daniels B, Lee CK, Simes RJ (2021). Cardiac assessment in Australian patients receiving (neo)adjuvant trastuzumab for HER2-positive early breast cancer: a population-based study. Breast Cancer Res Treat.

[29] Tang M, Schaffer A, Kiely BE, Daniels B, Simes RJ, Lee CK (2019). Treatment patterns and survival in HER2-positive early breast cancer: a whole-of-population Australian cohort study (2007–2016). Br J Cancer.

[30] Gannon MR, Dodwell D, Jauhari Y, Horgan K, Clements K, Medina J (2020). Initiation of adjuvant chemotherapy and trastuzumab for human epidermal growth receptor 2-positive early invasive breast cancer in a population-based cohort study of older women in England. J Geriatr Oncol.

[31] von Elm E, Altman DG, Egger M, Pocock SJ, Gøtzsche PC, Vandenbroucke JP (2007). The strengthening the reporting of observational studies in epidemiology (STROBE) statement: guidelines for reporting observational studies. Ann Intern Med.

[32] Henson KE, Elliss-Brookes L, Coupland VH, Payne E, Vernon S, Rous B (2020). Data resource profile: national cancer registration dataset in England. Int J Epidemiol.

[33] Office for National Statistics England: detailed information on the administrative structure within England.

[34] Union for International Cancer Control (2016). TNM: classification of malignant tumours.

[35] Bright CJ, Lawton S, Benson S, Bomb M, Dodwell D, Henson KE (2020). Data resource profile: the systemic anti-cancer therapy (SACT) dataset. Int J Epidemiol.

[36] Fraser J, Wills L, Fardus-Reid F, Irvine L, Elliss-Brookes L, Fern L (2021). Oral etoposide as a single agent in childhood and young adult cancer in England: still a poorly evaluated palliative treatment. Pediatr Blood Cancer.

[37] Wallington M, Saxon EB, Bomb M, Smittenaar R, Wickenden M, McPhail S (2016). 30-day mortality after systemic anticancer treatment for breast and lung cancer in England: a population-based, observational study. Lancet Oncol.

[38] All.Can Systemic anti-cancer therapy (SACT) data set: collecting and using real-world data to improve cancer care.

[39] Department for Communities and Local Government (2015). The English index of multiple deprivation (IMD).

[40] Jerrim J (2023). Measuring parental income using administrative data. What is the best proxy available?. Res Pap Educ.

[41] National Cancer Registration Analysis Service Collecting and using data.

[42] Bibby P, Brindley P The 2011 rural-urban classification for small area geographies: a user guide and frequently asked questions (v10).

[43] Charlson ME, Pompei P, Ales KL, MacKenzie CR (1987). A new method of classifying prognostic comorbidity in longitudinal studies: development and validation. J Chronic Dis.

[44] Du XL, Xia R, Burau K, Liu CC (2011). Cardiac risk associated with the receipt of anthracycline and trastuzumab in a large nationwide cohort of older women with breast cancer, 1998–2005. Med Oncol.

[45] Norris RP, Dew R, Greystoke A, Todd A, Sharp L (2023). Socio-economic inequalities in novel NSCLC treatments during the era of tumor biomarker guided therapy: a population-based cohort study in a publicly funded healthcare system. J Thorac Oncol.

[46] Woods LM, Rachet B, Morris M, Bhaskaran K, Coleman MP (2021). Are socio-economic inequalities in breast cancer survival explained by peri-diagnostic factors?. BMC Cancer.

[47] Benitez Majano S, Di Girolamo C, Rachet B, Maringe C, Guren MG, Glimelius B (2019). Surgical treatment and survival from colorectal cancer in Denmark, England, Norway, and Sweden: a population-based study. Lancet Oncol.

[48] Li R, Daniel R, Rachet B (2016). How much do tumor stage and treatment explain socioeconomic inequalities in breast cancer survival? Applying causal mediation analysis to population-based data. Eur J Epidemiol.

[49] McKeage K, Perry CM (2002). Trastuzumab: a review of its use in the treatment of metastatic breast cancer overexpressing HER2. Drugs.

[50] National Institute for Health and Care Excellence Recommendations, early and locally advanced breast cancer: diagnosis and management guidance.

[51] Link BG, Phelan J (1995). Social conditions as fundamental causes of disease. J Health Soc Behav.

[52] Sankar P, Cho MK, Condit CM, Hunt LM, Koenig B, Marshall P (2004). Genetic research and health disparities. JAMA.

[53] Mannion R, Thompson C (2014). Systematic biases in group decision-making: implications for patient safety. Int J Qual Health Care.

[54] Shore ND, Morgans AK, El-Haddad G, Srinivas S, Abramowitz M (2022). Addressing challenges and controversies in the management of prostate cancer with multidisciplinary teams. Target Oncol.

[55] Raine R, Wallace I, Nic a’ Bháird C, Xanthopoulou P, Lanceley A, Clarke A (2014). Improving the effectiveness of multidisciplinary team meetings for patients with chronic diseases: a prospective observational study. Health Services and Delivery Research.

[56] Puts MTE, Tapscott B, Fitch M, Howell D, Monette J, Wan-Chow-Wah D (2015). A systematic review of factors influencing older adults’ decision to accept or decline cancer treatment. Cancer Treat Rev.

[57] White M, Adams J, Heywood P, Babones SJ (2012). Social inequality and public health.

[58] Victora CG, Vaughan JP, Barros FC, Silva AC, Tomasi E (2000). Explaining trends in inequities: evidence from Brazilian child health studies. Lancet Lond Engl.

[59] National Institute for Health and Care Excellence Overview - guidance on the use of trastuzumab for the treatment of advanced breast cancer.

[60] National Institute for Health and Care Excellence Final appraisal determination trastuzumab for the adjuvant treatment of early-stage HER2-positive breast cancer.

[61] Piantadosi S, Byar DP, Green SB (1988). The ecological fallacy. Am J Epidemiol.

[62] Delon C, Brown KF, Payne NWS, Kotrotsios Y, Vernon S, Shelton J (2022). Differences in cancer incidence by broad ethnic group in England, 2013-2017. Br J Cancer.

[63] Schneeweiss S, Maclure M (2000). Use of comorbidity scores for control of confounding in studies using administrative databases. Int J Epidemiol.

[64] Fernandez ME, Ruiter RAC, Markham CM, Kok G (2019). Intervention mapping: theory and evidence-based health promotion program planning: perspective and examples. Front Public Health.

[65] Dharamsi S, Espinoza N, Cramer C, Amin M, Bainbridge L, Poole G (2010). Nurturing social responsibility through community service-learning: lessons learned from a pilot project. Med Teach.

[66] Dietrich AJ, Tobin JN, Cassells A, Robinson CM, Greene MA, Sox CH (2006). Telephone care management to improve cancer screening among low-income women: a randomized, controlled trial. Ann Intern Med.

[67] Metzger Filho O, Burstein HJ (2018). Duration of adjuvant trastuzumab: might less be more?. Ann Oncol.

