# Sociodemographic Disparities in HER2^+^ Breast Cancer Trastuzumab Receipt: An English Population-Based Study

**DOI:** 10.1158/1055-9965.EPI-24-0144

**Published:** 2024-07-15

**Authors:** Ruth P. Norris, Rosie Dew, Alastair Greystoke, Nicola Cresti, Henry Cain, Adam Todd, Linda Sharp

**Affiliations:** 1 Centre for Cancer, Population Health Sciences Institute, Newcastle University, Newcastle-upon-Tyne, United Kingdom.; 2 Northern Centre for Cancer Care, Freeman Hospital, Newcastle Hospital Trust, Newcastle-upon-Tyne, United Kingdom.; 3 School of Pharmacy, Newcastle University, Newcastle-upon-Tyne, United Kingdom.

## Abstract

**Background:**

Sociodemographic disparities in traditional breast cancer treatment receipt in nonpublicly funded healthcare systems are well documented. This study investigated trastuzumab receipt by sociodemographic factors within a female, HER2^+^ breast cancer population in England’s publicly funded National Health Service.

**Methods:**

The English national population-based cancer registry and linked Systemic Anti-Cancer Therapy database identified 36,985 women with HER2^+^ invasive breast cancer diagnosed between January 1, 2012 and December 31, 2017. Multivariable logistic regression determined the likelihood of trastuzumab receipt in early and metastatic disease by the deprivation category of area of residence and other sociodemographic characteristics.

**Results:**

Early-stage trastuzumab receipt followed a socioeconomic gradient. Women residing in the most deprived areas were 10% less likely to receive trastuzumab [multivariable OR 0.90; 95% confidence interval (CI), 0.83–0.98] compared with women residing in the least deprived areas. In both early and metastatic disease, trastuzumab receipt was less likely in older women with more comorbidities, estrogen receptor–positive disease, and who were not discussed at a multidisciplinary team meeting.

**Conclusions:**

Despite the provision of free care at the point of delivery in England, sociodemographic disparities in early-stage HER2^+^ trastuzumab receipt occur. Further research determining how inequities contribute to disparities in outcomes is warranted to ensure optimized trastuzumab use for all.

**Impact:**

Fair access to novel cancer treatments regardless of place of residence, sociodemographic characteristics, and/or cancer stage requires prioritization in future cancer improvement policies.

*See related In the Spotlight, p. 1259*

## Introduction

Over the past decades, patients with breast cancer have benefitted from the use of novel, targeted anticancer therapies (1). The mAb trastuzumab, targeting the HER2 in patients with HER2 overexpressed/amplified (HER2^+^) breast cancer (approximately 20% of breast cancer diagnoses worldwide; ref. 2) provides a clear example (3). Trastuzumab has extended treatment choice beyond traditional treatments (surgery and cytotoxic chemotherapy; ref. 4), and this has improved prognosis in an aggressive breast cancer subtype (5). Although treatment of HER2^+^ breast cancer is increasingly personalized and evolving (6, 7), trastuzumab remains a crucial care component, offering women a 33% reduction in breast cancer mortality in early stage disease [ratio of annual death rates = 0.67, 95% confidence interval (CI), 0.61–0.73; ref. 8] and an 18% reduction in overall survival in women with metastatic disease (pooled HR = 0.82; 95% CI, 0.71–0.94; ref. 9).

Breast cancer is subject to socioeconomic and sociodemographic disparities. Increased incidence is associated with higher socioeconomic status (SES; ref. 10), whereas higher mortality and reduced survival are linked to women with a lower SES, perhaps in part due to barriers related to treatment access (11). Lower SES has historically been associated with reduced receipt of traditional breast cancer treatments, including breast conserving surgery (12), neoadjuvant chemotherapy (e.g., anthracyclines and taxanes; ref. 13), and radiotherapy (12). In addition, access to breast cancer treatment varies by ethnicity, health insurance status, and geographic location (14, 15). However, this body of research has tended to combine all breast cancer subtypes. It is less clear, therefore, whether HER2^+^-specific treatment, in particular targeted and historically high-cost treatments such as trastuzumab—which may be hypothesized to be more frequently received by the economically advantaged (i.e., those with private finance and/or insurance)—are also subject to differences in receipt by SES and wider sociodemographic factors (16).

Real-world evidence documenting socioeconomic disparities in novel breast cancer treatment receipt is emerging. For example, a recent meta-analysis concluded that a low SES is associated with lower novel anticancer therapy receipt across a range of cancers (including trastuzumab use in HER2^+^ breast cancer; ref. 17). Similar findings have subsequently been reported in recent observational studies (18, 19). In addition, sociodemographic and clinical inequities in trastuzumab receipt are highlighted in an older systematic review of observational studies; receipt was higher in women who were younger, had fewer comorbidities, a higher tumor grade, a larger tumor size, an advanced stage cancer, and a negative hormone receptor status (20). However, previous population-based studies have predominantly reported US data; trastuzumab receipt in a publicly funded healthcare system has seldom been reported (17). There are some data from the mixed Chinese (21, 22) and Indian (23) healthcare systems as well as the publicly funded Canadian and Australian health systems (24–29), but these studies are few in number or include comparatively small cohorts of treated patients only (with no denominator populations precluding calculation of odds of receipt). The UK National Health Service (NHS) provides an example of a nationwide publicly funded healthcare system in which trastuzumab access is free at the point of delivery to all patients, and clinical guidelines are biomarker-driven. The only available UK data reported to date have examined trastuzumab initiation in older women in the context of adjuvant chemotherapy receipt (30). It, therefore, remains unclear whether socioeconomic disparities in trastuzumab receipt occur in healthcare systems in which individual finance and/or insurance are not considered a factor, and whether such inequities are present in patients of all ages, and with both early and metastatic disease.

To investigate, a large population-based observational study was undertaken using NHS data in England. The aim of this study was to determine the association of SES (measured using the deprivation category at the area of residence) and wider sociodemographic characteristics with receipt of trastuzumab in a stage I–III (early) and stage IV (metastatic) HER2^+^ invasive breast cancer female population using data from a publicly funded healthcare system.

## Materials and Methods

### Study design and setting

Population-based data were extracted from the National Cancer Registry Database (NCRD) and Systemic Anti-Cancer Therapy (SACT) dataset in England for all cases of women, of any age, diagnosed with a primary invasive stage I–IV breast tumor (International Classification of Diseases (ICD), Tenth revision C50.0–C50.9) between January 1, 2012, and December 31, 2017. Favorable ethical approval was obtained from the Proportionate Review Sub-committee of the West Midlands-Edgbaston Research Ethics Committee on October 16, 2019 (ref 19/WM/0317). Section 251 of the NHS Act 2006 grants legal permission to register information on diagnosed cancers without the need to seek patient consent. The study was performed in accordance with the Declaration of Helsinki and is reported according to the Strengthening the Reporting of Observational Studies in Epidemiology guidelines (Supplementary Materials and Methods; ref. 31).

### Data sources and linkage

The NCRD is a national cancer registry for patients living in England, diagnosed with malignant and premalignant neoplasms (32). Registry data are compiled into an event-based registration model with patient NHS numbers providing unique identifiers for data linkage (32). NCRD data obtained were as follows: deprivation category of area of residence at diagnosis measured using quintile rank of the income domain of the Index of Multiple Deprivation (IMD), sex, age, year of diagnosis, ethnicity, rural/urban residence, government region (33), stage at diagnosis (tumor, nodes, and metastasis summary stage; ref. 34), grade, HER2 status, estrogen receptor (ER) status, presence of multiple tumors, number of comorbidities, discussion at a multidisciplinary team (MDT) meeting, and receipt of cancer directed surgery and/or chemotherapy within 6 months of diagnosis.

SACT is a relatively new resource, capturing drug level information on routinely administered SACT (e.g., standard chemotherapy, targeted therapy, immunotherapy, targeted biologicals, and modifying supportive therapies) in secondary and tertiary NHS providers in England (35). Data collection started in April 2012 and by April 2014, monthly NHS hospital trust submissions were mandated (36, 37). The SACT data guide treatment delivery and inform the National Institute for Health and Care Excellence’s (NICE) drug funding decisions, though other uses (e.g., audits, research provision, drug monitoring, and clinical trial data follow up) exist (35, 38). SACT data, linked to cancer registrations, provided information on trastuzumab receipt.

### Study population

The population of interest was women diagnosed with stage I–IV HER2^+^ breast cancer (defined as 3+ HER2 staining on IHC or HER2 amplification using *in situ* hybridization if 2+ on IHC). To manage instances of multiple primary breast tumor registrations in patients, a hierarchy determined which tumor record to retain for analysis: (i) earliest diagnosis; (ii) most advanced stage at diagnosis; (iii) most specific ICD code (ICD C50.0-C50.8); or (iv) first tumor entry. Males were excluded as male breast cancer is rare (*n* = 1,815). Further exclusions included tumors with negative or unknown HER2 status (*n* = 221,299) and tumors with a stage at diagnosis of 0 and/or unknown stage (*n* = 3,293). This left an analytical cohort of 36,985 patients (stage I–III, *n* = 34,616; stage IV, *n* = 2,369; Supplementary Figs. S1 and S2).

### Outcome variable

The primary focus was trastuzumab receipt, recorded as a binary (Y/N) outcome variable. A patient who had a SACT record with a reference to receipt of trastuzumab (either alone or in combination with other drugs) was categorized as receiving trastuzumab (Y). Currently, SACT data lack treatment indication detail; hence, a timeframe restriction of 56 days prior to and 1-year post diagnosis date was applied to increase confidence that trastuzumab use was for the primary invasive HER2^+^ breast cancer of interest. Supplementary Table S1 provides a breakdown of trastuzumab SACT data codes included for this outcome variable.

### Explanatory variables

The main explanatory variable was the deprivation category (proxy SES measure). In the NCRD, the IMD provides an area-based measure of relative deprivation for each small area (containing an average of 1,500 people) assigned based on the postcode of residence at the time of cancer diagnosis (39). IMD is a widely used composite index for classifying SES in England, based on the characteristics of small areas (40). Although IMD is derived from seven domains (income, employment, education, skills and training, health deprivation and disability, crime, barriers to housing and services, and living environment), the NCRD only makes available the income domain (proportion of the population experiencing deprivation relating to low income— taken as both those out of work as well as those in work but who have low earnings; ref. 41). The IMD income domain (henceforth referred to as IMD) was grouped into quintiles (1, least deprived; 5, most deprived). As IMD is updated periodically, the status closest to the date of breast cancer diagnosis was applied (i.e., IMD 2010 for diagnosis in 2012 and IMD 2015 for those diagnosed 2013 to 2017). Additional sociodemographic variables of interest were age at diagnosis, ethnic group, rural/urban residence, and government region. Other potential covariates considered were stage at diagnosis, tumor grade, presence of multiple tumors, number of comorbidities, ER status, whether women received surgery and/or chemotherapy within 6 months of diagnosis, whether each case was discussed at MDT meeting, and year of diagnosis. Age was categorized into <50, 50 to 59, 60 to 69, 70 to 79, and 80+ years old. Ethnicity was classified as White, other ethnic group (Asian/British, Asian, Black/African/Caribbean/Black British, mixed/multiple ethic groups, and other ethnic groups), and unknown (missing and unknown classifications). Rural/urban residence was defined as rural village, hamlet, and isolated dwellings; rural town and fringe; urban city and town; and urban conurbation (42). The following nine government regions were used: North West, North East, West Midlands, Yorkshire and the Humber, East Midlands, East of England, South East, South West, and London (33). Tumor, nodes, and metastasis stage at diagnosis was categorized as I, II, III, and IV. Grade was grouped as well, moderately, and poorly differentiated, and other (undifferentiated, anaplastic, undetermined, or missing tumor grade). The multiple tumors’ variable took the value of 1 if the index breast cancer was the only cancer the individual had and was more than 1 if they had previously had (an)other cancer(s). ER status was classified as positive (at least one positive test, including borderline definitions), negative, or unknown. The number of comorbidities was determined from a weighted Charlson Comorbidity Index score (43), applied to conditions (with the exception of the index cancer) that resulted in hospital admissions in the period 78 to 6 months prior to diagnosis, and was categorized none, 1 to 2, and 3+. The receipt of surgery and chemotherapy within 6 months of cancer diagnosis was categorized as yes or no. Discussion at MDT meeting was classified as yes, no, or missing. Finally, the year of diagnosis explored temporal associations in treatment receipt.

### Statistical analysis

Baseline demographic and clinical characteristics (number and percentage) were summarized for the full study cohort (stages I–IV). Descriptive statistics (number and percentage) are listed by all independent variables of interest for both the early (stages I–III) and metastatic (stage IV) sub-populations. χ^2^ tests determined associations between sociodemographic/clinical characteristics and trastuzumab receipt in these two populations.

The likelihood of trastuzumab receipt in the early-stage and metastatic populations, by deprivation and all other sociodemographic/clinical characteristics, was determined with univariable and multivariable logistic regression models. Any significant clinical and demographic variables in univariable analyses [likelihood ratio test (LRT) ≤0.05] were included in multivariate models. Deprivation, as the primary variable of interest, was forced into all models with IMD 1 (least deprived) used as the reference group. Models report unadjusted and adjusted multivariable ORs (mvOR) with 95% CI(s) and *P* values. Model fit was checked, using Hosmer and Lemeshow χ^2^ tests, and variables contributing to poor fit were excluded. The Akaike information criterion assisted decision-making in instances in which selection between competing models was needed. Variance inflation factors were computed to provide an additional collinearity check; final model variables all had variance inflation factors <10. Throughout, a *P* value of ≤0.05 (two-sided tests) was considered statistically significant. In final multivariable models, a test for linear trend across deprivation categories was calculated.

Sensitivity analyses limited the stage I–III and stage IV cohorts to patients with date of incidence from April 2014 onward to reflect the period when SACT reporting by hospital trusts became mandatory (sensitivity analysis 1) and a refined (more definitive) HER2 status classification (i.e., positive only) to minimize the possibility of misclassification (sensitivity analysis 2).

All statistical analyses were conducted using Stata version 16.1 (StataCorp, College Station, Texas).

### Data availability

The data analyzed in this study are available from the current data controller, NHS England. Restrictions apply to the availability of these data, which were used under the license for this study only; the authors are not permitted to share these data.

## Results

### Patient characteristics

A total of 36,985 patients were diagnosed with stage I–IV HER2^+^ breast cancer between January 1, 2012, and December 31, 2017. Most women were of White ethnicity (88.1%); just under half were aged 50 to 69 (49.2%), a similar percentage resided in urban cities and towns (45.3%), and most had no comorbidities (82.3%). Much of the cohort had stage I–III tumors (93.6%); more than 90% graded as moderate or poorly differentiated (92.7%); and almost two-thirds as ER-positive (63.7%). Population demographic and clinical characteristics are shown in Table 1.

**Table 1.** Demographic and clinical characteristics of women with stage I–IV HER2^+^ breast cancer diagnosed between January 01, 2012 and December 31, 2017 (*n* = 36,985).

	Stages I–IV36,985 (100.00)	Stages I–III34,616 (93.59)	Stage IV 2,369 (6.41)
Characteristic	Number (%)		
Deprivation^a^			
1 (least deprived)	8,454 (22.86)	7,970 (23.02)	484 (20.43)
2	8,267 (22.35)	7,786 (22.49)	481 (20.30)
3	7,469 (20.19)	7,024 (20.29)	445 (18.78)
4	6,814 (18.42)	6,299 (18.20)	515 (21.74)
5 (most deprived)	5,981 (16.17)	5,537 (16.00)	444 (18.74)
Age at diagnosis (years)			
<50	8,962 (24.23)	8,466 (24.46)	496 (20.94)
50–59	9,132 (24.69)	8,617 (24.89)	515 (21.74)
60–69	9,058 (24.49)	8,592 (24.82)	466 (19.67)
70–79	5,997 (16.21)	5,489 (15.86)	508 (21.44)
80+	3,836 (10.37)	3,452 (9.97)	384 (16.21)
Ethnicity			
White	32,565 (88.05)	30,500 (88.11)	2,065 (87.17)
Other ethnic group^b^	2,852 (7.71)	2,653 (7.66)	199 (8.40)
Unknown^c^	1,568 (4.24)	1,463 (4.23)	105 (4.43)
Rural/urban residence			
Rural village, hamlet, and isolated dwellings	4,008 (10.84)	3,772 (10.90)	236 (9.96)
Rural town and fringe	3,942 (10.66)	3,686 (10.65)	256 (10.81)
Urban city and town	16,744 (45.27)	15,666 (45.26)	1,078 (45.50)
Urban conurbation	12,291 (33.23)	11,492 (33.20)	799 (33.73)
Government region			
North West	5,937 (16.05)	5,583 (16.13)	354 (14.94)
North East	2,437 (6.59)	2,287 (6.61)	150 (6.33)
West Midlands	3,929 (10.62)	3,683 (10.64)	246 (10.38)
Yorkshire and the Humber	3,310 (8.95)	3,056 (8.83)	254 (10.72)
East Midlands	3,128 (8.46)	2,958 (8.55)	170 (7.18)
East of England	5,010 (13.55)	4,666 (13.48)	344 (14.52)
South East	5,711 (15.44)	5,316 (15.36)	395 (16.67)
South West	4,049 (10.95)	3,836 (11.08)	213 (8.99)
London	3,474 (9.39)	3,231 (9.33)	243 (10.26)
Stage at diagnosis			
I	13,094 (35.40)	13,094 (37.83)	—
II	16,663 (45.05)	16,663 (48.14)	—
III	4,859 (13.14)	4,859 (14.04)	—
IV	2,369 (6.41)	—	2,369 (100.00)
Grade			
Well differentiated	2,034 (5.50)	1,999 (5.77)	35 (1.48)
Moderately differentiated	16,077 (43.47)	15,122 (43.69)	955 (40.31)
Poorly differentiated	18,217 (49.26)	16,989 (49.08)	1,228 (51.84)
Other^d^	657 (1.78)	506 (1.46)	151 (6.37)
Multiple tumors^e^			
1 tumor only	32,570 (88.06)	30,533 (88.20)	2,037 (85.99)
>1 tumors	4,415 (11.94)	4,083 (11.80)	332 (14.01)
ER status			
Positive^f^	23,557 (63.69)	22,299 (64.42)	1,258 (53.10)
Negative	7,912 (21.39)	7,233 (20.89)	679 (28.66)
Unknown^g^	5,516 (14.91)	5,084 (14.69)	432 (18.24)
Number of comorbidities (between 78 and 6 months prior to diagnosis)^h^			
None	30,423 (82.26)	28,494 (82.31)	1,929 (81.43)
1–2	5,369 (14.52)	5,016 (14.49)	353 (14.90)
3+	1,193 (3.23)	1,106 (3.20)	87 (3.67)
Discussed at MDT meeting			
Yes	25,997 (70.29)	24,672 (71.27)	1,325 (55.93)
No	5,789 (15.65)	5,352 (15.46)	437 (18.45)
Missing	5,199 (14.06)	4,592 (13.27)	607 (25.62)
Diagnosis year			
2012	4,099 (11.08)	3,810 (11.01)	289 (12.20)
2013	4,982 (13.47)	4,638 (13.40)	344 (14.52)
2014	5,705 (15.43)	5,325 (15.38)	380 (16.04)
2015	6,561 (17.74)	6,144 (17.75)	417 (17.60)
2016	7,476 (20.21)	7,031 (20.31)	445 (18.78)
2017	8,162 (22.07)	7,668 (22.15)	494 (20.85)
Treatment^i^			
Received chemotherapy^j^^,^^k^	22,893 (61.90)	21,353 (61.69)	1,540 (65.01)
Received surgery^j^^,^^k^	28,738 (77.70)	28,294 (81.74)	444 (18.74)
Received trastuzumab^l^	16,629 (44.96)	15,567 (44.97)	1,062 (44.83)

a Refers to IMD (income domain). For diagnosis year 2012, IMD_2010 was used; and for diagnosis years 2013 to 2017, IMD_2015 was used.

b Other ethnic group refers to Asian/British Asian, Black/African/Caribbean/Black British, mixed/multiple ethnic groups and other ethnic groups.

c Unknown ethnicity refers to unknown and missing ethnicity classifications.

d Other grade refers to undifferentiated, anaplastic, undetermined, and missing tumor grades.

e Refers to the number of tumors other than the index breast cancer (1 tumor only).

f Refers to at least one positive ER test and includes borderline definitions.

g Refers to unknown, not performed, or missing ER status.

h Refers to Charlson Comorbidity Index.

i Not exclusive, so do not total analytical cohort totals.

j Within 6 months of diagnosis. Surgery receipt beyond 6 months (e.g., following neoadjuvant chemotherapy) not captured.

k Data from NCRD.

l Data from SACT.

### Trastuzumab receipt: early-stage population

Of the 34,616 women with early stage HER2^+^ disease diagnosed between 2012 and 2017, 45.0% (*n* = 15,567) received trastuzumab. Receipt increased over time from 34.1% of patients in 2012 to 44.2% in 2017. In univariate analyses, trastuzumab receipt showed little patterning by SES. However, following adjustment for confounders (including clinical factors) in the multivariable model, a significant association between trastuzumab receipt and deprivation was seen (LRT = 0.004). Patients residing in the most deprived areas were 10% less likely to receive trastuzumab than those residing in the least deprived areas (IMD 5 vs. IMD 1; mvOR 0.90; 95% CI, 0.83–0.98; Table 2). The test for linear trend across deprivation categories was significant (*P* = 0.002).

**Table 2.** Early-stage disease: likelihood (OR with 95% CI and *P* values from logistic regression) of receiving trastuzumab by deprivation and adjusted for age, ethnicity, rural/urban residence, government region, stage at diagnosis, whether received surgery, ER status, comorbidities, whether discussed at MDT meeting, and diagnosis year for women with stage I–III HER2^+^ breast cancer who were diagnosed between January 01, 2012 and December 31, 2017 (*n* = 34,616).

				Unadjusted			Adjusted		
	Number (%) receivingtrastuzumab *n* = 15,567 (44.97)	Number (%) not receiving trastuzumab*n* = 19,049 (55.03)	*P* value^a^	OR	95% CI	*P* value^b^	OR	95% CI	*P* value^b^
Deprivation^c^			0.021			**0.020**			**0.004**
1 (least deprived)	3,605 (45.23)	4,365 (54.77)		1.00	—	—	1.00	—	—
2	3,525 (45.27)	4,261 (54.73)		1.00	0.94–1.07	0.958	1.00	0.94–1.08	0.910
3	3,039 (43.27)	3,985 (56.73)		0.92	0.87–0.99	0.016	0.90	0.84–0.96	0.003
4	2,847 (45.20)	3,452 (54.80)		1.00	0.93–1.07	0.967	0.93	0.86–1.00	0.050
5 (most deprived)	2,551 (46.07)	2,986 (53.93)		1.03	0.97–1.11	0.335	0.90	0.83–0.98	0.016
Age at diagnosis (years)			<0.001			**<0.001**			**<0.001**
<50	5,167 (61.03)	3,299 (38.97)		1.00	—	—	1.00	—	—
50–59	4,625 (53.67)	3,992 (46.33)		0.74	0.70–0.79	<0.001	0.79	0.74–0.84	<0.001
60–69	3,912 (45.53)	4,680 (54.47)		0.53	0.50–0.58	<0.001	0.59	0.55–0.63	<0.001
70–79	1,676 (30.53)	3,813 (69.47)		0.28	0.26–0.30	<0.001	0.27	0.25–0.29	<0.001
80+	187 (5.42)	3,265 (94.58)		0.04	0.03–0.04	<0.001	0.03	0.03–0.03	<0.001
Ethnicity			<0.001			**<0.001**			**<0.001**
White	13,720 (44.98)	16,780 (55.02)		1.00	—	—	1.00	—	—
Other ethnic group^d^	1,393 (52.51)	1,260 (47.49)		1.35	1.25–1.46	<0.001	0.93	0.84–1.02	0.110
Unknown^e^	454 (31.03)	1,009 (68.97)		0.55	0.49–0.62	<0.001	0.47	0.41–0.53	<0.001
Rural/urban residence			<0.001			**<0.001**			**0.000**
Rural village, hamlet, and isolated dwellings	1,688 (44.75)	2,084 (55.25)		1.05	0.97–1.12	0.218	1.03	0.95–1.12	0.466
Rural town and fringe	1,627 (44.14)	2,059 (55.86)		1.02	0.95–1.10	0.584	1.07	0.98–1.16	0.121
Urban city and town	6,837 (43.64)	8,829 (56.36)		1.00	—	—	1.00	—	—
Urban conurbation	5,415 (47.12)	6,077 (52.88)		1.15	1.10–1.21	<0.001	1.16	1.09–1.25	<0.001
Government region			<0.001			**<0.001**			**<0.001**
North West	2,509 (44.94)	3,074 (55.06)		1.00	—	—	1.00	—	—
North East	897 (39.22)	1,390 (60.78)		0.79	0.72–0.87	<0.001	0.70	0.63–0.79	<0.001
West Midlands	1,543 (41.90)	2,140 (58.10)		0.88	0.81–0.96	0.004	0.83	0.75–0.91	<0.001
Yorkshire and the Humber	1,687 (55.20)	1,369 (44.80)		1.51	1.38–1.65	<0.001	1.41	1.28–1.56	<0.001
East Midlands	1,395 (47.16)	1,563 (52.84)		1.09	1.00–1.20	0.050	1.09	0.99–1.21	0.090
East of England	1,983 (42.50)	2,683 (57.50)		0.91	0.84–0.98	0.013	0.88	0.80–0.97	0.008
South East	2,346 (44.13)	2,970 (55.87)		0.97	0.90–1.04	0.396	1.00	0.92–1.10	0.945
South West	1,685 (43.93)	2,151 (56.07)		0.96	0.88–1.04	0.331	1.07	0.97–1.18	0.186
London	1,522 (47.11)	1,709 (52.89)		1.09	1.00–1.19	0.049	0.92	0.83–1.03	0.139
Stage at diagnosis			<0.001			**<0.001**			**<0.001**
I	4,680 (35.74)	8,414 (64.26)		1.00	—	—	1.00	—	
II	8,189 (49.14)	8,474 (50.86)		1.74	1.66–1.82	<0.001	1.91	1.81–2.01	<0.001
III	2,698 (55.53)	2,161 (44.47)		2.24	2.10–2.40	<0.001	2.57	2.38–2.77	<0.001
Grade			<0.001			**<0.001**			—^l^
Well differentiated	232 (11.61)	1,767 (88.39)		0.10	0.09–0.12	<0.001	—	—	—
Moderately differentiated	5,575 (36.87)	9,547 (63.13)		0.46	0.44–0.48	<0.001	—	—	—
Poorly differentiated	9,533 (56.11)	7,456 (43.89)		1.00	—	—	—	—	—
Other^f^	227 (44.86)	279 (55.14)		0.64	0.53–0.76	<0.001	—	—	—
Received surgery within 6 months of diagnosis^g^			<0.001			**<0.001**			**<0.001**
Yes	12,536 (44.31)	15,758 (55.69)		1.00	—	—	1.00	—	—
No	3,031 (47.94)	3,291 (52.06)		1.16	1.10–1.22	<0.001	1.36	1.27–1.45	<0.001
Multiple tumors^h^			<0.001			**<0.001**			—^l^^l^
1 tumor only	14,135 (46.29)	16,398 (53.71)		1.00	—	—	—	—	—
>1 tumors	1,432 (35.07)	2,651 (64.93)		0.63	0.58–0.67	<0.001	—	—	—
ER status			<0.001			**<0.001**			**<0.001**
Positive^i^	8,859 (39.73)	13,440 (60.27)		1.00	—	—	1.00	—	—
Negative	4,204 (58.12)	3,029 (41.88)		2.11	2.00–2.22	<0.001	2.29	2.16–2.43	<0.001
Unknown^j^	2,504 (49.25)	2,580 (50.75)		1.47	1.39–1.57	<0.001	1.42	1.33–1.53	<0.001
Number of comorbidities (between 78 and 6 months prior to diagnosis)^k^			<0.001			**<0.001**			**<0.001**
0	13,614 (47.78)	14,880 (52.22)		1.00	—	—	1.00	—	—
1–2	1,776 (35.41)	3,240 (64.59)		0.60	0.56–0.64	<0.001	0.83	0.77–0.89	<0.001
3+	177 (16.00)	929 (84.00)		0.21	0.18–0.24	<0.001	0.38	0.32–0.46	<0.001
Discussed at MDT meeting			<0.001			**<0.001**			**<0.001**
Yes	11,584 (46.95)	13,088 (53.05)		1.00	—	—	1.00	—	—
No	2,222 (41.52)	3,130 (58.48)		0.80	0.76–0.85	<0.001	0.81	0.75–0.87	<0.001
Missing	1,761 (38.35)	2,831 (61.65)		0.70	0.66–0.75	<0.001	0.56	0.52–0.60	<0.001
Diagnosis year			<0.001			**<0.001**			**<0.001**
2012	1,299 (34.09)	2,511 (65.91)		0.65	0.60–0.71	<0.001	0.50	0.46–0.55	<0.001
2013	2,155 (46.46)	2,483 (53.54)		1.10	1.02–1.18	0.013	0.94	0.86–1.02	0.128
2014	2,521 (47.34)	2,804 (52.66)		1.14	1.06–1.22	<0.001	1.04	0.96–1.13	0.300
2015	2,892 (47.07)	3,252 (52.93)		1.12	1.05–1.20	0.001	1.10	1.02–1.19	0.012
2016	3,314 (47.13)	3,717 (52.87)		1.13	1.06–1.20	<0.001	1.15	1.07–1.23	<0.001
2017	3,386 (44.16)	4,282 (55.84)		1.00	—	—	1.00	—	—

a *χ* ^2^ *P* value.

b Bolded *P* values are from LRT of the variable’s contribution to the model. Unbolded *P* values are from a test of whether the OR is different from 1.

c Refers to IMD (income domain). For diagnosis year 2012, IMD_2010 was used and for diagnosis years 2013 to 2017, IMD_2015 was used.

d Other ethnic group refers to Asian/British Asian, Black/African/Caribbean/Black British, mixed/multiple ethnic groups, and other ethnic groups.

e Unknown ethnicity refers to missing and unknown ethnicity classifications.

f Other grade refers to undifferentiated, anaplastic, undetermined, and missing tumor grades.

g Surgery receipt beyond 6 months (e.g., following neoadjuvant chemotherapy) not captured.

h Refers to the number of tumors other than the index breast cancer (1 tumor only).

i Refers to at least one positive ER test and includes borderline definitions.

j Refers to unknown, not performed, or missing ER status.

k Defined using the Charlson Comorbidity Index.

l Variable not included in the adjusted analysis as resulted in poor model fit (assessed using Hosmer and Lemeshow *χ*^2^ test).

In sensitivity analyses, similar associations between trastuzumab receipt and deprivation were observed when restricting analyses to a HER2^+^ breast cancer diagnosis after mandatory SACT submission (April 2014; sensitivity analysis 1; IMD 5 vs. IMD 1; mvOR 0.93; 95% CI, 0.84–1.03; LRT *P* = 0.009) and were more defined when the refined HER2^+^ breast cancer definition (sensitivity analysis 2) was applied (IMD 5 vs. IMD 1; mvOR 0.86; 95% CI, 0.78–0.95; LRT *P* = 0.009). Both results were statistically significant (Supplementary Table S2).

Several other sociodemographic and clinical variables in the multivariable model also had statistically significant associations with reduced likelihood of trastuzumab receipt. These were an older age (80+ vs. <50 years old; mvOR 0.03; 95% CI, 0.03–0.03); three or more comorbidities (3+ vs. 0 comorbidities; mvOR 0.38; 95% CI, 0.32–0.46); and not being discussed at MDT meeting (no vs. yes; mvOR 0.81; 95% CI, 0.75–0.87). The ethnic group made a statistically significant contribution to the model. A negative ER status (negative vs. positive; mvOR 2.29; 95% CI, 2.16–2.43), a higher stage cancer (stage III vs. stage I; mvOR 2.57; 95% CI, 2.38–2.77), and not receiving surgery (no vs. yes; mvOR 1.36; 95% CI, 1.27–1.45) were all associated with increased trastuzumab receipt. There was no patterning of trastuzumab receipt with urban/rural residence (Table 2).

### Trastuzumab receipt: metastatic population

Of the 2,369 women with metastatic breast HER2^+^ breast cancer diagnosed between 2012 and 2017, 44.8% (*n* = 1,062) received trastuzumab. Receipt increased over time, from 29.1% of patients diagnosed in 2012 to 51.0% of patients diagnosed in 2017. In univariable and multivariable analyses, trastuzumab receipt did not vary significantly by deprivation quintile of residence (multivariable analysis, LRT *P* = 0.225) even following adjustment for clinical factors, or have a significant linear trend (*P* = 0.864). Odds of receipt followed a u-shaped pattern, being slightly below unity for deprivation quintiles 2 to 4, and reaching borderline significance for the middle category (IMD 3 vs. IMD 1; mvOR 0.75; 95% CI, 0.56–1.00; Table 3).

**Table 3.** Metastatic disease: likelihood (OR with 95% CI and *P* values from logistic regression) of receiving trastuzumab by deprivation and adjusted for age, ethnicity, rural/urban residence, government region, grade, whether received surgery, ER status, comorbidities, whether discussed at MDT meeting, and diagnosis year for women with stage IV HER2^+^ breast cancer diagnosed between January 01, 2012 and December 31, 2017 (*n* = 2,369).

				Unadjusted			Adjusted		
	Number (%) receivingtrastuzumab *n* = 1,062 (44.83)	Number (%) not receiving trastuzumab *n* = 1,307 (55.17)	*P* value^a^	OR	95% CI	*P* value^b^	OR	95% CI	*P* value^b^
Deprivation^c^			0.314			**0.312**			**0.225**
1 (least deprived)	224 (46.28)	260 (53.72)		1.00	—	—	1.00	—	—
2	215 (44.70)	266 (55.30)		0.94	0.73–1.21	0.622	0.85	0.64–1.13	0.270
3	182 (40.90)	263 (59.10)		0.80	0.62–1.04	0.099	0.75	0.56–1.00	0.052
4	229 (44.47)	286 (55.53)		0.93	0.72–1.19	0.565	0.84	0.63–1.12	0.232
5 (most deprived)	212 (47.75)	232 (52.25)		1.06	0.82–1.37	0.655	1.00	0.74–1.37	0.977
Age at diagnosis (years)			<0.001			**<0.001**			**<0.001**
<50	314 (63.31)	182 (36.69)		1.00	—	—	1.00	—	—
50–59	285 (55.34)	230 (44.66)		0.72	0.56–0.92	0.010	0.67	0.51–0.87	0.003
60–69	223 (47.85)	243 (52.15)		0.53	0.41–0.69	<0.001	0.55	0.41–0.73	<0.001
70–79	171 (33.66)	337 (66.34)		0.29	0.23–0.38	<0.001	0.27	0.20–0.36	<0.001
80+	69 (17.97)	315 (82.03)		0.13	0.09–0.17	<0.001	0.11	0.08–0.16	<0.001
Ethnicity			<0.001			**<0.001**			**0.002**
White	923 (44.70)	1,142 (55.30)		1.00	—	—	1.00	—	—
Other ethnic group^d^	109 (54.77)	90 (45.23)		1.50	1.12–2.01	0.007	1.00	0.71–1.41	0.998
Unknown^e^	30 (28.57)	75 (71.43)		0.49	0.32–0.76	0.001	0.44	0.27–0.71	0.001
Rural/urban residence			0.042			**0.042**			**0.010**
Rural village, hamlet, and isolated dwellings	116 (49.15)	120 (50.85)		1.31	0.99–1.74	0.058	1.48	1.06–2.04	0.020
Rural town and fringe	107 (41.80)	149 (58.20)		0.98	0.74–1.29	0.862	1.07	0.79–1.46	0.665
Urban city and town	457 (42.39)	621 (57.61)		1.00	—	—	1.00	—	—
Urban conurbation	382 (47.81)	417 (52.19)		1.24	1.04–1.50	0.020	1.45	1.11–1.87	0.005
Government region			0.003			**0.002**			**<0.001**
North West	170 (48.02)	184 (51.98)		1.00	—	—	1.00	—	—
North East	63 (42.00)	87 (58.00)		0.78	0.53–1.15	0.215	0.60	0.39–0.93	0.023
West Midlands	85 (34.55)	161 (65.45)		0.57	0.41–0.80	0.001	0.38	0.26–0.56	<0.001
Yorkshire and the Humber	130 (51.18)	124 (48.82)		1.13	0.82–1.57	0.442	0.84	0.58–1.21	0.350
East Midlands	89 (52.35)	81 (47.65)		1.19	0.82–1.72	0.354	0.95	0.62–1.46	0.815
East of England	143 (41.57)	201 (58.43)		0.77	0.57–1.04	0.087	0.68	0.48–0.99	0.042
South East	185 (46.84)	210 (53.16)		0.95	0.72–1.27	0.745	0.81	0.57–1.15	0.235
South West	88 (41.31)	125 (58.69)		0.76	0.54–1.07	0.121	0.84	0.56–1.27	0.418
London	109 (44.86)	134 (55.14)		0.88	0.63–1.22	0.446	0.56	0.38–0.83	0.004
Grade			<0.001			**<0.001**			**0.030**
Well differentiated	8 (22.86)	27 (77.14)		0.30	0.14–0.67	0.003	0.33	0.14–0.78	0.011
Moderately differentiated	386 (40.42)	569 (59.58)		0.69	0.58–0.82	0.000	0.86	0.71–1.04	0.117
Poorly differentiated	609 (49.59)	619 (50.41)		1.00	—	—	1.00	—	—
Other^f^	59 (39.07)	92 (60.93)		0.65	0.46–0.92	0.015	0.87	0.59–1.29	0.491
Received surgery within 6 months of diagnosis^g^			<0.001			**0.000**			**0.016**
Yes	234 (52.70)	210 (47.30)		1.48	1.20–1.82	<0.001	1.33	1.05–1.69	0.016
No	828 (43.01)	1,097 (56.99)		1.00	—	—	1.00	—	—
Multiple tumors^h^			0.001			**0.001**			—^l^^l^
1 tumor only	941 (46.20)	1,096 (53.80)		1.00	—	—	—	—	—
>1 tumors	121 (36.45)	211 (63.55)		0.67	0.53–0.85	0.001	—	—	—
ER status			<0.001			**<0.001**			**<0.001**
Positive^i^	482 (38.31)	776 (61.69)		1.00	—	—	1.00	—	—
Negative	367 (54.05)	312 (45.95)		1.89	1.57–2.29	<0.001	2.17	1.74–2.69	<0.001
Unknown^j^	213 (49.31)	219 (50.69)		1.57	1.26–1.95	<0.001	1.49	1.16–1.93	0.002
Number of comorbidities (between 78 and 6 months prior to diagnosis)^k^			<0.001			**<0.001**			**0.015**
0	906 (46.97)	1,023 (53.03)		1.00	—	—	1.00	—	—
1–2	136 (38.53)	217 (61.47)		0.71	0.56–0.89	0.004	0.94	0.72–1.22	0.634
3+	20 (22.99)	67 (77.01)		0.34	0.20–0.56	<0.001	0.46	0.26–0.80	0.006
Discussed at MDT meeting			0.001			**0.001**			**<0.001**
Yes	637 (48.08)	688 (51.92)		1.00	—	—	1.00	—	—
No	188 (43.02)	249 (56.98)		0.82	0.66–1.01	0.067	0.78	0.60–1.00	0.053
Missing	237 (39.04)	370 (60.96)		0.69	0.57–0.84	<0.001	0.57	0.46–0.72	<0.001
Diagnosis year			<0.001			**<0.001**			**<0.001**
2012	84 (29.07)	205 (70.93)		0.39	0.29–0.54	<0.001	0.26	0.19–0.37	<0.001
2013	143 (41.57)	201 (58.43)		0.68	0.52–0.90	0.007	0.47	0.34–0.65	<0.001
2014	163 (42.89)	217 (57.11)		0.72	0.55–0.94	0.017	0.52	0.38–0.70	<0.001
2015	204 (48.92)	213 (51.08)		0.92	0.71–1.19	0.529	0.76	0.56–1.02	0.066
2016	216 (48.54)	229 (51.46)		0.91	0.70–1.17	0.449	0.82	0.61–1.09	0.163
2017	252 (51.01)	242 (48.99)		1.00	—	—	1.00	—	—

Abbreviations: n, number.

a *χ* ^2^ *P* value.

b Bolded *P* values are from LRT of the variable’s contribution to the model. Unbolded *P* values are from a test of whether the OR is different from 1.

c Refers to IMD (income domain). For diagnosis year 2012, IMD_2010 was used and for diagnosis years 2013 to 2017, IMD_2015 was used.

d Other ethnic group refers to Asian/British Asian, Black/African/Caribbean/Black British, mixed/multiple ethnic groups, and other ethnic groups.

e Unknown ethnicity refers to missing and unknown ethnicity classifications.

f Other grade refers to undifferentiated, anaplastic, undetermined, and missing tumor grades.

g Surgery receipt beyond 6 months (e.g., following neoadjuvant chemotherapy) not captured.

h Refers to the number of tumors other than the index breast cancer (1 tumor only).

i Refers to at least one positive ER test and includes borderline definitions.

j Refers to unknown, not performed, or missing ER status.

k Defined using the Charlson Comorbidity Index.

l Variable not included in the adjusted analysis as resulted in poor model fit (assessed using Hosmer and Lemeshow *χ*^2^ test).

In sensitivity analyses, when restricting consideration to breast cancer diagnosis after mandatory SACT submission (April 2014; sensitivity analysis 1), the univariable ORs were similar to the primary analysis, but the multivariable model did not have adequate fit (Supplementary Table S3). Restriction of the analysis to a refined HER2^+^ breast cancer definition (sensitivity analysis 2) showed no significant associations between IMD and trastuzumab receipt (Supplementary Table S3).

Several other demographic and clinical variables in the multivariable model had statistically significant associations with reduced likelihood of trastuzumab receipt in stage IV patients. These were an older age (80+ vs. <50 years old; mvOR 0.11; 95% CI, 0.08–0.16); a well differentiated tumor grade (well vs. poorly differentiated grade; mvOR 0.33; 95% CI, 0.14–0.78); three or more comorbidities (3+ vs. 0 comorbidities; mvOR 0.46; 95% CI, 0.26–0.80); and no discussion at MDT meeting (no vs. yes; mvOR; 0.78; 95% CI, 0.60–1.00). Trastuzumab receipt was more common in women with ER-negative tumors (negative vs. positive; mvOR; 2.17; 95% CI, 1.74–2.69). Additionally, trastuzumab receipt was associated with both a White and non-White ethnicity (other ethnic group vs. White ethnicity; mvOR; 1.00; 95% CI, 0.71–1.41) as well as residence in urban conurbations (urban conurbation vs. urban city and town; mvOR 1.45; 95% CI, 1.11–1.87) and rural villages (rural village vs. urban city and town; mvOR 1.48; 95% CI, 1.06–2.04; Table 3).

## Discussion

This study addresses associations between IMD and other sociodemographic and clinical characteristics with trastuzumab receipt among a cohort of patients with HER2^+^ breast cancer in England during 2012 to 2017. It represents one of the few nationwide studies using SACT data and is the largest study internationally of trastuzumab receipt in a publicly funded healthcare system [and the second largest study on this topic after Du and colleagues (44), which reported US trastuzumab data up to 2005]. This study found that women residing in areas of greater deprivation with early-stage HER2^+^ breast cancer were 10% less likely to receive trastuzumab than women residing in the least deprived areas. No clear or statistically significant associations between SES and trastuzumab receipt were found in the metastatic HER2^+^ cohort. A younger age was associated with increased trastuzumab receipt for all patients. Other associations of increased trastuzumab receipt were seen, in patients with both early and metastatic disease, who had fewer comorbidities, an ER negative tumor, and who were discussed at MDT meeting. For early-stage disease, a higher staged tumor and not receiving surgery within 6 months increased the likelihood of trastuzumab receipt. However, for metastatic disease, a moderate or poorly differentiated tumor grade and receiving surgery within 6 months was associated with increased trastuzumab receipt.

This population-based study significantly strengthens the evidence base showing that socioeconomic disparities of HER2^+^ breast cancer treatment occur even in healthcare systems in which targeted therapy is free at the point of access. Our parallel study on socioeconomic inequalities in NSCLC treatments in England over the same time period also found that greater deprivation was associated with reduced novel therapy use, though the magnitude of associations was starker (45). Given that socioeconomic differences in treatment receipt may account for disparities in cancer survival and mortality (46–48), these inequities have important implications.

There is no clear, single explanation as to why trastuzumab receipt is less commonly used in women with early-stage breast cancer residing in more deprived areas or, as shown here, why receipt varies by age or rural–urban residence. Trastuzumab is well-tolerated, with toxicity being lower than that of cytotoxic chemotherapy therapy, and monitoring is in place to minimize cardiovascular complications (49). Theoretically, many women (regardless of socioeconomic background) should tolerate trastuzumab. One barrier to receipt is HER2 testing access. However, as the NIH and Care Excellence recommends HER2 testing in England as routine (50), testing is well-established in this cancer. It is possible that women residing in areas of high deprivation, or older women, maybe more likely to decline HER2 testing when offered. However, data from a previous systematic review and meta-analysis found only a small reduction in HER2 testing access by low SES, and this was not statistically significant (17).

An alternative explanation for socioeconomic (and, indeed, wider sociodemographic) patterning of trastuzumab receipt maybe that novel treatment analyses fail to consider the “fundamental causes” or “upstream” factors (i.e., unequal distribution of income and education) which generate inequity (51). Trastuzumab receipt is a “downstream intervention” focused on the final stages of the care pathway. The role of wider social determinants of health, which also influence inequality generation and persistence, is not addressed through trastuzumab licensing (52). Furthermore, MDTs may be an additional source of socioeconomic biases (conscious or unconscious) in treatment decision-making (53). MDT discussion was important for increasing the likelihood of trastuzumab receipt, despite the fact that all accredited breast cancer units managing patients in England should be considered at MDT meeting. This suggests that MDTs facilitate evidence-based, standardized clinical decision-making around trastuzumab use (54). Previous work has discussed how the implementation of MDT decision can vary by deprivation status (55); however, more work is needed to explore whether MDTs mitigate socioeconomic biases in targeted treatment access. Finally, patient views of, and willingness to accept, trastuzumab maybe socioeconomically/sociodemographically patterned. Treatment involves returning regularly to the hospital for up to a year; this may be challenging and/or less appealing to the oldest patients or those with limited economic resources. Specific research on trastuzumab treatment decision-making is lacking; however, a 2015 systematic review reported generally that convenience and transportation difficulties were key determinants of older adults’ decisions to accept or decline cancer treatment (56).

Socioeconomic associations in trastuzumab receipt varied by cancer stage, with an association evident for early stage, but not for metastatic disease. Potentially this reflects time since licensing. New drug interventions may become intervention-generated inequality (IGI) examples when their introduction preferentially benefits those of higher SES with resources to gain priority access (57). IGIs are particularly concerning when treatments are new and wane over time as interventions become “standard practice” (inverse inequity hypothesis; ref. 58). Minimal socioeconomic disparities in metastatic compared with early-stage trastuzumab receipt may reflect first licensing in metastatic HER2^+^ disease in England in 2002 (access widened to early-stage breast cancer in 2006; refs. 59, 60). This hypothesis cannot be considered nationally as SACT was only established in 2012.

This study is amongst the first to report English population registry–based data analyzing the receipt of a high-cost targeted treatment (trastuzumab) and exploring an emerging big data resource (SACT) with a focus on socioeconomic disparities. The national dataset coverage minimizes selection bias, improves data completeness, and enhances study validity. Despite these strengths, there are several limitations. First, early SACT data completeness prior to mandated trust submission after April 2014 is uncertain and may explain apparent low overall trastuzumab receipt (37). However, sensitivity analyses for early-stage disease confirmed that associations with deprivation were not impacted by time. Second, NCRD data collection across healthcare providers can vary (35). This may explain why surgery rates in early-stage breast cancer were lower than anticipated. Third, it is possible that recording of trastuzumab by hospital trusts is biased by deprivation category or other sociodemographic factors. However, given the comparability in demographic characteristics between patients both with and without SACT information recorded (not shown) and the fact that hospital catchment areas have diverse populations, this seems unlikely. Fourth, IMD was measured at the area level rather than the patient level and only considered a single domain of deprivation (income), so care is needed to avoid the ecological fallacy and the assumption that similar associations would be observed with other SES measures, especially those at the individual level (e.g., education level and employment status; ref. 61). Moreover, ethnicity was based on information recorded in hospital records; although the quality of ethnicity data for the period of the study is considered better than in earlier years (62), 4.2% of patients were recorded as “unknown” ethnic group (and only 7.7% as the non-White ethnic group), which may have introduced misclassification. Combining non-White ethnicities into one group for the purpose of analysis meant that the variation between the constituent ethnic groups could not be investigated; this, in turn, limits the ability to target interventions tackling inequalities based on ethnicity. Fifth, it was not possible to account for all factors serving as a barrier to treatment receipt (e.g., a low performance status). Sixth, although comorbidity presence was adjusted for in models, the Charlson Comorbidity Index (computed from hospital admissions in the period 78 to 6 months prior to cancer diagnosis) is a crude measure, so there is likely residual confounding by fitness for treatment (63). Finally, this study reports a snapshot of trastuzumab receipt pre-COVID-19 pandemic in one country. Results may not be generalizable to other novel high-cost targeted treatments, countries, or the period since 2017.

Future research has several priorities: (i) exploring whether inequities in trastuzumab receipt explain observed disparities in outcomes (survival and quality of life); (ii) seeking to better understand “causal mechanisms” underpinning current findings; and (iii) extending inequity evaluations to other novel therapies (including those in which predictive biomarker testing is not undertaken) and other cancers. From a policy and practice perspective, an increasing focus on implementing effective strategies and policies to overcome unfair novel treatment access is needed. This is pertinent given that targeted treatments are expanding for HER2 and other breast cancer subtypes (e.g., abemaciclib). Timely monitoring of novel treatment receipt to ensure that inequities do not become established is needed. Solutions likely require attention from patient, NHS provider, healthcare system, and wider society levels. The application of approaches like Intervention Mapping (a framework that uses theory and evidence to support intervention development) would be of value to inform the systematic development and testing of solutions. As an initial step, improved understanding of the determinants of utilization is required (64). Later stages could consider adaption of interventions successfully applied in other contexts (65, 66) to improve medication utilization. Examples include targeted health literacy interventions to improve patient participation in shared treatment decision-making, increased education of clinicians in the social determinants of health, and use of patient navigators to support more disadvantaged patients. Finally, interventions, which may improve the ease of trastuzumab use (e.g., shorter duration of therapy; ref. 67), could also be of value in reducing potential socioeconomic barriers (e.g., financial).

There are sociodemographic disparities in the receipt of trastuzumab for HER2^+^ breast cancer in England. In both early and metastatic disease, older women with more comorbidities, ER positive disease, and who are not discussed at MDT meeting are less likely to receive trastuzumab. Reduced trastuzumab receipt among women residing in more deprived areas was also observed in those with early-stage disease. These inequities are present, despite biomarker-driven guidelines and trastuzumab being free at the point of delivery in the publicly funded NHS. National policies to address inequalities in trastuzumab and other novel breast cancer treatments are urgently needed. Policies should focus on ensuring fair access regardless of place of residence, sociodemographic characteristics, and/or cancer stage.

## Supplementary Material

Supplementary Figure S1 Early-stage Disease Analytical Cohort Flow Diagram Supplementary Figure S1 Early-stage Disease Analytical Cohort Flow Diagram_Clean

Supplementary Figure S2 Metastatic Disease Analytical Cohort Flow Diagram Supplementary Figure S2 Metastatic Disease Analytical Cohort Flow Diagram_Clean

Supplementary Table S1 Trastuzumab coding references in the SACT dataset Supplementary Table S1 Trastuzumab coding references in the SACT dataset_Clean

Supplementary Table S2 Early-stage Disease Trastuzumab Receipt Sensitivity Analysis Supplementary Table S2 Early-stage Disease Trastuzumab Receipt Sensitivity Analysis_Clean

Supplementary Table S3 Metastatic Disease Trastuzumab Receipt Sensitivity Analysis Supplementary Table S3 Metastatic Disease Trastuzumab Receipt Sensitivity Analysis_Clean

Supplementary Materials and Methods Supplementary Materials and Methods STROBE Checklist_Clean

## References

[1] Crimini E , Repetto M, Aftimos P, Botticelli A, Marchetti P, Curigliano G. Precision medicine in breast cancer: from clinical trials to clinical practice. Cancer Treat Rev 2021;98:102223. 34049187 10.1016/j.ctrv.2021.102223

[2] Arteaga CL , Sliwkowski MX, Osborne CK, Perez EA, Puglisi F, Gianni L. Treatment of HER2 positive breast cancer: current status and future perspectives. Nat Rev Clin Oncol 2011;9:16–32. 22124364 10.1038/nrclinonc.2011.177

[3] Hudis CA . Trastuzumab-mechanism of action and use in clinical practice. N Engl J Med 2007;357:39–51. 17611206 10.1056/NEJMra043186

[4] Slamon D , Pegram M. Rationale for trastuzumab (Herceptin) in adjuvant breast cancer trials. Semin Oncol 2001;28(1 Suppl 3):13–9. 10.1016/s0093-7754(01)90188-5 11301370

[5] Slamon DJ , Clark GM, Wong SG, Levin WJ, Ullrich A, McGuire WL. Human breast cancer: correlation of relapse and survival with amplification of the HER-2/neu oncogene. Science 1987;235:177–82. 3798106 10.1126/science.3798106

[6] Cardoso F , Kyriakides S, Ohno S, Penault-Llorca F, Poortmans P, Rubio IT, . Early breast cancer: ESMO Clinical Practice Guidelines for diagnosis, treatment and follow-up. Ann Oncol 2019;30:1194–220. 31161190 10.1093/annonc/mdz173

[7] Gennari A , André F, Barrios CH, Cortés J, de Azambuja E, DeMichele A, . ESMO Clinical Practice Guideline for the diagnosis, staging and treatment of patients with metastatic breast cancer. Ann Oncol 2021;32:1475–95. 34678411 10.1016/j.annonc.2021.09.019

[8] Bradley R , Braybrooke J, Gray R, Hills R, Liu Z, Peto R, . Trastuzumab for early-stage, HER2-positive breast cancer: a meta-analysis of 13,864 women in seven randomised trials. Lancet Oncol 2021;22:1139–50. 34339645 10.1016/S1470-2045(21)00288-6 PMC8324484

[9] Balduzzi S , Mantarro S, Guarneri V, Tagliabue L, Pistotti V, Moja L, . Trastuzumab-containing regimens for metastatic breast cancer. Cochrane Database Syst Rev 2014;2014:CD006242. 24919460 10.1002/14651858.CD006242.pub2 PMC6464904

[10] Van Loon AJ , Goldbohm RA, Van den Brandt PA. Socioeconomic status and breast cancer incidence: a prospective cohort study. Int J Epidemiol 1994;23:899–905. 7860169 10.1093/ije/23.5.899

[11] Lagerlund M , Bellocco R, Karlsson P, Tejler G, Lambe M. Socio-economic factors and breast cancer survival- a population-based cohort study (Sweden). Cancer Causes Control 2005;16:419–30. 15953984 10.1007/s10552-004-6255-7

[12] Dreyer MS , Nattinger AB, McGinley EL, Pezzin LE. Socioeconomic status and breast cancer treatment. Breast Cancer Res Treat 2018;167:1–8. 28884392 10.1007/s10549-017-4490-3 PMC5790605

[13] Neuner JM , Kong A, Blaes A, Riley D, Chrischilles E, Smallwood A, . The association of socioeconomic status with receipt of neoadjuvant chemotherapy. Breast Cancer Res Treat 2019;173:179–88. 30232683 10.1007/s10549-018-4954-0 PMC6687292

[14] Singh S , Sridhar P. A narrative review of sociodemographic risk and disparities in screening, diagnosis, treatment, and outcomes of the most common extrathoracic malignancies in the United States. J Thorac Dis 2021;13:3827–43. 34277073 10.21037/jtd-21-87 PMC8264686

[15] All Party Parliamentary Group on Breast Cancer . A mixed picture: an inquiry into geographical inequalities and breast cancer. [cited 2023 Nov 29]. Available from: https://breastcancernow.org/sites/default/files/appgbc_a_mixed_picture.pdf.

[16] Tuckson RV , Newcomer L, de Sa JM. Accessing genomic medicine: affordability, diffusion, and disparities. JAMA 2013;309:1469–70. 23571584 10.1001/jama.2013.1468

[17] Norris RP , Dew R, Sharp L, Greystoke A, Rice S, Johnell K, . Are there socio-economic inequalities in utilization of predictive biomarker tests and biological and precision therapies for cancer? A systematic review and meta-analysis. BMC Med 2020;18:282. 33092592 10.1186/s12916-020-01753-0 PMC7583194

[18] Kaufman PA , Hurvitz SA, O’Shaughnessy J, Mason G, Yardley DA, Brufsky AM, . Baseline characteristics and first-line treatment patterns in patients with HER2-positive metastatic breast cancer in the SystHERs registry. Breast Cancer Res Treat 2021;188:179–90. 33641083 10.1007/s10549-021-06103-z

[19] Nguy S , Wu SP, Oh C, Gerber NK. Outcomes of HER2-positive non-metastatic breast cancer patients treated with anti-HER2 therapy without chemotherapy. Breast Cancer Res Treat 2021;187:815–30. 33590386 10.1007/s10549-021-06115-9

[20] Martin AP , Downing J, Cochrane M, Collins B, Francis B, Haycox A, . Trastuzumab uptake in HER2-positive breast cancer patients: a systematic review and meta-analysis of observational studies. Crit Rev Oncol Hematol 2018;130:92–107. 30196916 10.1016/j.critrevonc.2018.07.012

[21] Diao Y , Lin M, Xu K, Huang J, Wu X, Li M, . How government health insurance coverage of novel anti-cancer medicines benefited patients in China - a retrospective analysis of hospital clinical data. BMC Health Serv Res 2021;21:856. 34419013 10.1186/s12913-021-06840-3 PMC8380313

[22] Shang L , Lin Y, Fang W, Liu Y, Bao Y, Li X, . How national health insurance coverage policy affected the use of trastuzumab and rituximab in China: a bicentric retrospective study. Risk Manag Healthc Policy 2023;16:1739–53. 37692767 10.2147/RMHP.S420899 PMC10488736

[23] Singh P , Pasricha R, Pandey L, Joseph D, Ibrahim A, Bhadoria AS, . Socioeconomic factors affecting trastuzumab usage in patients with breast cancer in a resource constrained setting in north India. Int J Community Med Public Health 2021;8:279–85.

[24] Kumachev A , Trudeau ME, Chan KKW. Associations among socioeconomic status, patterns of care and outcomes in breast cancer patients in a universal health care system: Ontario’s experience. Cancer 2016;122:893–8. 26696022 10.1002/cncr.29838

[25] Goldhar HA , Yan AT, Ko DT, Earle CC, Tomlinson GA, Trudeau ME, . The temporal risk of heart failure associated with adjuvant trastuzumab in breast cancer patients: a population study. J Natl Cancer Inst 2016;108:djv301. 26476433 10.1093/jnci/djv301

[26] Thavendiranathan P , Abdel-Qadir H, Fischer HD, Camacho X, Amir E, Austin PC, . Breast cancer therapy-related cardiac dysfunction in adult women treated in routine clinical practice: a population-based cohort study. J Clin Oncol 2016;34:2239–46. 27091709 10.1200/JCO.2015.65.1505

[27] Lu CY , Srasuebkul P, Drew AK, Chen K, Ward RL, Pearson SA. Trastuzumab therapy in Australia: which patients with HER2+ metastatic breast cancer are assessed for cardiac function? Breast 2013;22:482–7. 23664254 10.1016/j.breast.2013.04.011

[28] Tang M , Schaffer AL, Kiely BE, Daniels B, Lee CK, Simes RJ, . Cardiac assessment in Australian patients receiving (neo)adjuvant trastuzumab for HER2-positive early breast cancer: a population-based study. Breast Cancer Res Treat 2021;187:893–902. 33616773 10.1007/s10549-021-06135-5

[29] Tang M , Schaffer A, Kiely BE, Daniels B, Simes RJ, Lee CK, . Treatment patterns and survival in HER2-positive early breast cancer: a whole-of-population Australian cohort study (2007–2016). Br J Cancer 2019;121:904–11. 31673103 10.1038/s41416-019-0612-5 PMC6889396

[30] Gannon MR , Dodwell D, Jauhari Y, Horgan K, Clements K, Medina J, . Initiation of adjuvant chemotherapy and trastuzumab for human epidermal growth receptor 2-positive early invasive breast cancer in a population-based cohort study of older women in England. J Geriatr Oncol 2020;11:836–42. 32007402 10.1016/j.jgo.2020.01.005

[31] von Elm E , Altman DG, Egger M, Pocock SJ, Gøtzsche PC, Vandenbroucke JP, . The Strengthening the Reporting of Observational Studies in Epidemiology (STROBE) statement: guidelines for reporting observational studies. Ann Intern Med 2007;147:573–7. 17938396 10.7326/0003-4819-147-8-200710160-00010

[32] Henson KE , Elliss-Brookes L, Coupland VH, Payne E, Vernon S, Rous B, . Data resource profile: national cancer registration dataset in England. Int J Epidemiol 2020;49:16–h. 31120104 10.1093/ije/dyz076 PMC7124503

[33] Office for National Statistics . England: detailed information on the administrative structure within England. [cited 2021 Aug 30]. Available from: https://www.ons.gov.uk/methodology/geography/ukgeographies/administrativegeography/england.

[34] Union for International Cancer Control . TNM: classification of malignant tumours. 8th ed. New York (NY): Wiley; 2016.

[35] Bright CJ , Lawton S, Benson S, Bomb M, Dodwell D, Henson KE, . Data resource profile: the Systemic Anti-Cancer Therapy (SACT) dataset. Int J Epidemiol 2020;49:15–l. 31340008 10.1093/ije/dyz137 PMC7426029

[36] Fraser J , Wills L, Fardus-Reid F, Irvine L, Elliss-Brookes L, Fern L, . Oral etoposide as a single agent in childhood and young adult cancer in England: still a poorly evaluated palliative treatment. Pediatr Blood Cancer 2021;68:e29204. 34227732 10.1002/pbc.29204

[37] Wallington M , Saxon EB, Bomb M, Smittenaar R, Wickenden M, McPhail S, . 30-day mortality after systemic anticancer treatment for breast and lung cancer in England: a population-based, observational study. Lancet Oncol 2016;17:1203–16. 27599138 10.1016/S1470-2045(16)30383-7 PMC5027226

[38] All.Can . Systemic anti-cancer therapy (SACT) data set: collecting and using real-world data to improve cancer care. [cited 2022 Nov 12]. Available from: https://www.all-can.org/efficiency-hub/systemic-anti-cancer-therapy-sact-data-set-collecting-and-using-real-world-data-to-improve-cancer-care/.

[39] Department for Communities and Local Government . The English index of multiple deprivation (IMD) 2015. [cited 2021 Aug 28]. Available from: https://assets.publishing.service.gov.uk/government/uploads/system/uploads/attachment_data/file/464430/English_Index_of_Multiple_Deprivation_2015_-_Guidance.pdf.

[40] Jerrim J . Measuring parental income using administrative data. What is the best proxy available? Res Pap Educ 2023;0:1–25.

[41] National Cancer Registration Analysis Service . Collecting and using data. [cited 2024 Apr 8]. Available from: http://ncin.org.uk/collecting_and_using_data/.

[42] Bibby P , Brindley P. The 2011 rural-urban classification for small area geographies: a user guide and frequently asked questions (v1.0). [cited 2022 May 19]. Available from: https://assets.publishing.service.gov.uk/government/uploads/system/uploads/attachment_data/file/239478/RUC11user_guide_28_Aug.pdf.

[43] Charlson ME , Pompei P, Ales KL, MacKenzie CR. A new method of classifying prognostic comorbidity in longitudinal studies: development and validation. J Chronic Dis 1987;40:373–83. 3558716 10.1016/0021-9681(87)90171-8

[44] Du XL , Xia R, Burau K, Liu CC. Cardiac risk associated with the receipt of anthracycline and trastuzumab in a large nationwide cohort of older women with breast cancer, 1998–2005. Med Oncol 2011;28:80–90. 10.1007/s12032-010-9717-7 20967512

[45] Norris RP , Dew R, Greystoke A, Todd A, Sharp L. Socio-economic inequalities in novel NSCLC treatments during the era of tumor biomarker guided therapy: a population-based cohort study in a publicly funded healthcare system. J Thorac Oncol 2023;18:990–1002. 37146751 10.1016/j.jtho.2023.04.018

[46] Woods LM , Rachet B, Morris M, Bhaskaran K, Coleman MP. Are socio-economic inequalities in breast cancer survival explained by peri-diagnostic factors? BMC Cancer 2021;21:485. 33933034 10.1186/s12885-021-08087-x PMC8088027

[47] Benitez Majano S , Di Girolamo C, Rachet B, Maringe C, Guren MG, Glimelius B, . Surgical treatment and survival from colorectal cancer in Denmark, England, Norway, and Sweden: a population-based study. Lancet Oncol 2019;20:74–87. 30545752 10.1016/S1470-2045(18)30646-6 PMC6318222

[48] Li R , Daniel R, Rachet B. How much do tumor stage and treatment explain socioeconomic inequalities in breast cancer survival? Applying causal mediation analysis to population-based data. Eur J Epidemiol 2016;31:603–11. 27165500 10.1007/s10654-016-0155-5 PMC4956701

[49] McKeage K , Perry CM. Trastuzumab: a review of its use in the treatment of metastatic breast cancer overexpressing HER2. Drugs 2002;62:209–43. 11790161 10.2165/00003495-200262010-00008

[50] National Institute for Health and Care Excellence . Recommendations, early and locally advanced breast cancer: diagnosis and management guidance. [cited 2022 Mar 30]. Available from: https://www.nice.org.uk/guidance/ng101/chapter/Recommendations#surgery-to-the-breast.

[51] Link BG , Phelan J. Social conditions as fundamental causes of disease. J Health Soc Behav 1995;Spec No:80–94. 7560851

[52] Sankar P , Cho MK, Condit CM, Hunt LM, Koenig B, Marshall P, . Genetic research and health disparities. JAMA 2004;291:2985–9. 15213210 10.1001/jama.291.24.2985 PMC2271142

[53] Mannion R , Thompson C. Systematic biases in group decision-making: implications for patient safety. Int J Qual Health Care 2014;26:606–12. 25320152 10.1093/intqhc/mzu083

[54] Shore ND , Morgans AK, El-Haddad G, Srinivas S, Abramowitz M. Addressing challenges and controversies in the management of prostate cancer with multidisciplinary teams. Target Oncol 2022;17:709–25. 36399218 10.1007/s11523-022-00925-7 PMC9672595

[55] Raine R , Wallace I, Nic a’ Bháird C, Xanthopoulou P, Lanceley A, Clarke A, . Improving the effectiveness of multidisciplinary team meetings for patients with chronic diseases: a prospective observational study. In: Health services and delivery research. Southampton (UK): NIHR Journals Library; 2014. 25642498

[56] Puts MTE , Tapscott B, Fitch M, Howell D, Monette J, Wan-Chow-Wah D, . A systematic review of factors influencing older adults’ decision to accept or decline cancer treatment. Cancer Treat Rev 2015;41:197–215. 25579752 10.1016/j.ctrv.2014.12.010

[57] White M , Adams J, Heywood P. How and why do interventions that increase health overall widen inequalities within populations? In: Babones SJ, editor. Social inequality and public health. Bristol: Policy Press; 2012. p. 64–81.

[58] Victora CG , Vaughan JP, Barros FC, Silva AC, Tomasi E. Explaining trends in inequities: evidence from Brazilian child health studies. Lancet Lond Engl 2000;356:1093–8. 10.1016/S0140-6736(00)02741-0 11009159

[59] National Institute for Health and Care Excellence . Overview - guidance on the use of trastuzumab for the treatment of advanced breast cancer. [cited 2021 Sep 17]. Available from: https://www.nice.org.uk/guidance/ta34.

[60] National Institute for Health and Care Excellence . Final appraisal determination trastuzumab for the adjuvant treatment of early-stage HER2-positive breast cancer. [cited 2021 Sep 16]. Available from: https://www.nice.org.uk/guidance/ta107/documents/final-appraisal-determination-breast-cancer-early-trastuzumab2.

[61] Piantadosi S , Byar DP, Green SB. The ecological fallacy. Am J Epidemiol 1988;127:893–904. 3282433 10.1093/oxfordjournals.aje.a114892

[62] Delon C , Brown KF, Payne NWS, Kotrotsios Y, Vernon S, Shelton J. Differences in cancer incidence by broad ethnic group in England, 2013–2017. Br J Cancer 2022;126:1765–73. 35233092 10.1038/s41416-022-01718-5 PMC9174248

[63] Schneeweiss S , Maclure M. Use of comorbidity scores for control of confounding in studies using administrative databases. Int J Epidemiol 2000;29:891–8. 11034974 10.1093/ije/29.5.891

[64] Fernandez ME , Ruiter RAC, Markham CM, Kok G. Intervention mapping: theory and evidence-based health promotion program planning: perspective and examples. Front Public Health 2019;7:209. 31475126 10.3389/fpubh.2019.00209 PMC6702459

[65] Dharamsi S , Espinoza N, Cramer C, Amin M, Bainbridge L, Poole G. Nurturing social responsibility through community service-learning: lessons learned from a pilot project. Med Teach 2010;32:905–11. 21039101 10.3109/01421590903434169

[66] Dietrich AJ , Tobin JN, Cassells A, Robinson CM, Greene MA, Sox CH, . Telephone care management to improve cancer screening among low-income women: a randomized, controlled trial. Ann Intern Med 2006;144:563–71. 16618953 10.7326/0003-4819-144-8-200604180-00006 PMC3841972

[67] Metzger Filho O , Burstein HJ. Duration of adjuvant trastuzumab: might less be more? Ann Oncol 2018;29:2273–4. 30357308 10.1093/annonc/mdy480

