# Supplementary material for: Sociodemographic Disparities in HER2+ Breast Cancer Trastuzumab Receipt: An English Population-Based Study
Source: Cancer Epidemiol Biomarkers Prev. 2024 Jul 15;33(10):1298–310. doi: 10.1158/1055-9965.EPI-24-0144 (PMC7616541; doi:10.1158/1055-9965.EPI-24-0144)
Supplement: Supplementary Figure S1 Early-stage Disease Analytical Cohort Flow Diagram — _Clean [file epi-24-0144_suppsf1.docx]

**Supplementary Figure S1** Early-stage disease analytical cohort flow diagram

^a^Negative and unknown HER2 status (the latter includes missing values n=53,157).

^b^Refers to stage at diagnosis.

^c^All analyses of trastuzumab receipt refer to receipt within the time range of interest. Those who had a record outside this time range have been grouped with “No SACT record” in subsequent analyses.

Abbreviations: HER2: Human epidermal growth factor receptor 2; IMD: Index of Multiple Deprivation (income domain); SACT: Systemic anti-cancer therapy (SACT).

This figure describes the derivation of the analysis cohort from the original dataset, specifying categories and numbers of exclusions. The number of patients utilizing trastuzumab, who had a SACT record administered with the time range of interest, is also shown.
