# Supplementary material for: Sociodemographic Disparities in HER2+ Breast Cancer Trastuzumab Receipt: An English Population-Based Study
Source: Cancer Epidemiol Biomarkers Prev. 2024 Jul 15;33(10):1298–310. doi: 10.1158/1055-9965.EPI-24-0144 (PMC7616541; doi:10.1158/1055-9965.EPI-24-0144)
Supplement: Supplementary Table S1 Trastuzumab coding references in the SACT dataset — _Clean [file epi-24-0144_supplementary_table_s1_suppst1.docx]

**Supplementary Table S1** Trastuzumab coding references in the SACT dataset.

| **SACT Drug Classification** | **SACT Analysis Group Listings** | **SACT Benchmark Group Listings** | **SACT Drug Group Listings** |
| --- | --- | --- | --- |
| TRASTUZUMAB  Anti-HER2 Targeted Therapy (mAb) | CAPECITABINE + **TRASTUZUMAB**  CARBOPLATIN + DOCETAXEL + PERTUZUMAB + **TRASTUZUMAB**  CARBO + DOCETAXEL + **TRASTUZUMAB**  CARBOPLATIN + DOCETAXEL + **TRASTUZUMAB**  CARBOPLATIN + PACLITAXEL + **TRASTUZUMAB**  CARBOPLATIN + **TRASTUZUMAB**  CYCLO + DOCETAXEL + **TRASTUZUMAB**  CYCLOPHOSPHAMIDE + DOCETAXEL + DOXORUBICIN + PERTUZUMAB + **TRASTZUMAB**  CYCLOPHOSPHAMIDE + DOCETAXEL + DOXORUBICIN + **TRASTUZUMAB**  CYCLOPHOSPHAMIDE + DOCETAXEL + PERTUZUMAB + **TRASTUZUMAB**  CYCLOPHOSPHAMIDE + DOCETAXEL + **TRASTUZUMAB**  CYCLOPHOSPHAMIDE + DOXORUBICIN + PERTUZUMAB + **TRASTUZUMAB**  DOCETAXEL + PERTUZUMAB + **TRASTUZUMAB**  DOCETAXEL + **TRASTUZUMAB**  EC + DOCETAXEL + **TRASTUZUMAB**  FEC + DOCETAXEL + PERTUZUMAB + **TRASTUZUMAB**  FEC + DOCETAXEL + **TRASTUZUMAB**  FEC 100 + DOCETAXEL + **TRASTUZUMAB**  FEC + PACLITAXEL + **TRASTUZUMAB**  FEC + **TRASTUZUMAB**  FEC 60 OR 75 + **TRASTUZUMAB**  FEC 100 + **TRASTUZUMAB**  LAPATINIB + **TRASTUZUMAB**  NAB-PACLITAXEL + **TRASTUZUMAB**  PACLITAXEL + PERTUZUMAB + **TRASTUZUMAB**  PACLITAXEL + **TRASTUZUMAB**  PERTUZUMAB + **TRASTUZUMAB**  TAC + **TRASTUZUMAB**  **T**CH  TRASTUZUMAB  **TRASTUZUMAB** + VINORELBINE  APHINITY TRIAL  BERENICE TRIAL  EPHOS-B TRIAL  METAPHER TRIAL  PERSEPHONE TRIAL  ROSCO TRIAL  SAFEHER TRIAL  SOLD TRIAL | CAPECITABINE + **TRASTUZUMAB**  CARBO + DOCETAXEL + **TRASTUZUMAB**  CARBOPLATIN + DOCETAXEL + PERTUZUMAB + **TRASTUZUMAB**  CARBOPLATIN + DOCETAXEL + **TRASTUZUMAB**  CARBOPLATIN + PACLITAXEL + **TRASTUZUMAB**  CARBOPLATIN + **TRASTUZUMAB**  CYCLO + DOCETAXEL + **TRASTUZUMAB**  CYCLOPHOSPHAMIDE + DOCETAXEL + DOXORUBICIN + PERTUZUMAB + **TRASTUZUMAB**  CYCLOPHOSPHAMIDE + DOCETAXEL + DOXORUBICIN + **TRASTUZUMAB**  CYCLOPHOSPHAMIDE + DOCETAXEL + PERTUZUMAB + **TRASTUZUMAB**  CYCLOPHOSPHAMIDE + DOCETAXEL + **TRASTUZUMAB**  CYCLOPHOSPHAMIDE + DOXORUBICIN + PERTUZUMAB + **TRASTUZUMAB**  DOCETAXEL + PERTUZUMAB + **TRASTUZUMAB**  DOCETAXEL + **TRASTUZUMAB**  EC + DOCETAXEL + **TRASTUZUMAB**  FEC + DOCETAXEL + PERTUZUMAB **+ TRASTUZUMAB**  FEC + DOCETAXEL + **TRASTUZUMAB**  FEC + PACLITAXEL + **TRASTUZUMAB**  FEC + **TRASTUZUMAB**  LAPATINIB + **TRASTUZUMAB**  NAB-PACLITAXEL + **TRASTUZUMAB**  PACLITAXEL + PERTUZUMAB + **TRASTUZUMAB**  PACLITAXEL + **TRASTUZUMAB**  PERTUZUMAB + **TRASTUZUMAB**  TAC + **TRASTUZUMAB**  **T**CH  TRASTUZUMAB  **TRASTUZUMAB** + VINORELBINE  APHINITY TRIAL  BERENICE TRIAL  EPHOS-B TRIAL  METAPHER TRIAL  PERSEPHONE TRIAL  ROSCO TRIAL  SAFEHER TRIAL  SOLD TRIAL | **TRASTUZUMAB**  **TRASTUZUMAB** (HERCEPTIN)  **TRASTUZUMAB** BIOSIMILAR (HERZUMA)  **TRASTUZUMAB** BIOSIMILAR (ONTRUZANT) |

Trials listed in table only if it is certain that all patients received trastuzumab.

Bold text indicates the reference to trastuzumab within the drug regimen.

This list defining trastuzumab regimens was compiled from therapies listed in the denominator registry population records. Clinical advice, along with evidence from NICE guidelines, the BNF, EMC, EMA, ASCO, and ESMO guidelines was then used to identify trastuzumab regimens during 2012-2017.

HER2 Trial Information

APHINITY Trial – Group 1 receives chemotherapy, trastuzumab and pertuzumab. Group 2 receives chemotherapy, trastuzumab and a placebo.

BERENICE Trial – Group 1 receives doxorubicin and cyclophosphamide then paclitaxel plus trastuzumab and pertuzumab. Group 2 receives fluorouracil, epirubicin, cyclophosphamide, then docetaxel with trastuzumab and pertuzumab.

EPHOS-B Trial – Everyone receives chemotherapy and trastuzumab after surgery. Group 1 have surgery, chemotherapy then trastuzumab. Group 2 have trastuzumab, before and after surgery, followed by chemotherapy then trastuzumab. Group 3 have lapatinib and trastuzumab before and after surgery followed by chemotherapy then trastuzumab.

MetaPHER Trial – Everyone receives docetaxel after trastuzumab and pertuzumab.

PERSEPHONE Trial – Everyone receives trastuzumab (either for 6 or 12 months) with or after chemotherapy.

ROSCO Trial – Either receive docetaxel and cyclophosphamide or FEC. If HER2+ also receive trastuzumab.

SafeHER Trial – Receive SC Trastuzumab via assisted administration conventional syringe and needle/vial formulation or with assisted or self-administration using a single-use injection device.

SOLD Trial – Everyone had docetaxel and trastuzumab at the same time to start then FEC. Some patients then received more trastuzumab.

Abbreviations: ASCO: American Society of Clinical Oncology; BNF: British National Formulary; EC: Epirubicin & Cyclophosphamide; EMA: European Medicines Agency; EMC: Electronic medicines compendium; ESMO: European Society for Medical Oncology; FEC: Fluorouracil, Epirubicin & Cyclophosphamide; mAb: Monoclonal Antibody; NICE: National Institute for Health and Care Research; SACT: Systemic Anti-Cancer Therapy; SC: subcutaneous; TAC: Docetaxel, Doxorubicin & Cyclophosphamide; TCH: Docetaxel, Carboplatin & Trastuzumab.
