# Supplementary material for: Sociodemographic Disparities in HER2+ Breast Cancer Trastuzumab Receipt: An English Population-Based Study
Source: Cancer Epidemiol Biomarkers Prev. 2024 Jul 15;33(10):1298–310. doi: 10.1158/1055-9965.EPI-24-0144 (PMC7616541; doi:10.1158/1055-9965.EPI-24-0144)
Supplement: Supplementary Table S2 Early-stage Disease Trastuzumab Receipt Sensitivity Analysis — _Clean [file epi-24-0144_supplementary_table_s2_suppst2.docx]

**Supplementary Table S2** Early-stage disease trastuzumab receipt: sensitivity analyses

|  |  |  |  | **Unadjusted** | | | **Adjusted** | | |
| --- | --- | --- | --- | --- | --- | --- | --- | --- | --- |
|  | Number (%)  Receiving  Trastuzumab | Number (%) Not Receiving Trastuzumab | P Value^a^ | OR | 95% CI | P Value^b^ | OR | 95% CI | P Value^b^ |
| ***Original Analysis: All Stage I-III Disease Diagnosed 2012-2017 (n = 34,616)*** | | |  |  |  |  |  |  |  |
|  |  |  |  |  |  |  |  |  |  |
| **Deprivation^c^** | n = 15,567 (44.97) | n = 19,049 (55.03) | 0.021 |  |  | **0.020** |  |  | **0.004** |
| 1 (Least Deprived) | 3,605 (45.23) | 4,365 (54.77) |  | 1.00 | ----- ----- | ------- | 1.00 | ----- ----- | ------- |
| 2 | 3,525 (45.27) | 4,261 (54.73) |  | 1.00 | 0.94 – 1.07 | 0.958 | 1.00 | 0.94 – 1.08 | 0.910 |
| 3 | 3,039 (43.27) | 3,985 (56.73) |  | 0.92 | 0.87 – 0.99 | 0.016 | 0.90 | 0.84 – 0.96 | 0.003 |
| 4 | 2,847 (45.20) | 3,452 (54.80) |  | 1.00 | 0.93 – 1.07 | 0.967 | 0.93 | 0.86 – 1.00 | 0.050 |
| 5 (Most Deprived) | 2,551 (46.07) | 2,986 (53.93) |  | 1.03 | 0.97 – 1.11 | 0.335 | 0.90 | 0.83 – 0.98 | 0.016 |
|  |  |  |  |  |  |  |  |  |  |
| ***Sensitivity Analysis 1: Diagnosis Date Post Mandated SACT Submission 01/04/2014 – 31/12/2017 (n = 24,826)*** | | | | | |  |  |  |  |
|  |  |  |  |  |  |  |  |  |  |
| **Deprivation^c^** | n = 11,495 (46.30) | n = 13,331 (53.70) | 0.043 |  |  | **0.042** |  |  | **0.009** |
| 1 (Least Deprived) | 2,629 (46.03) | 3,083 (53.97)) |  | 1.00 | ----- ----- | ------- | 1.00 | ----- ----- | ------- |
| 2 | 2,628 (46.97) | 2,967 (53.03) |  | 1.04 | 0.96 – 1.12 | 0.314 | 1.07 | 0.98 – 1.16 | 0.133 |
| 3 | 2,236 (44.55) | 2,783 (55.45) |  | 0.94 | 0.87 – 1.02 | 0.126 | 0.92 | 0.84 – 1.00 | 0.054 |
| 4 | 2,132 (46.75) | 2,428 (53.25) |  | 1.03 | 0.95 – 1.11 | 0.462 | 0.96 | 0.88 – 1.06 | 0.435 |
| 5 (Most Deprived) | 1,870 (47.46) | 2,070 (52.54) |  | 1.06 | 0.98 – 1.15 | 0.165 | 0.93 | 0.84 – 1.03 | 0.151 |
|  |  |  |  |  |  |  |  |  |  |
| ***Sensitivity Analysis 2: Positive HER2+ Status Definition (n = 23,533)*** | | |  |  |  |  |  |  |  |
|  |  |  |  |  |  |  |  |  |  |
| **Deprivation^c^** | n = 13,995 (59.47) | n = 9,538 (40.53) | 0.100 |  |  | **0.100** |  |  | **0.009** |
| 1 (Least Deprived) | 3,276 (59.96) | 2,188 (40.04) |  | 1.00 | ----- ----- | ------- | 1.00 | ----- ----- | ------- |
| 2 | 3,166 (59.43) | 2,161 (40.57) |  | 0.98 | 0.91 – 1.06 | 0.580 | 0.96 | 0.88 – 1.04 | 0.332 |
| 3 | 2,741 (57.88) | 1,995 (42.12) |  | 0.92 | 0.85 – 0.99 | 0.033 | 0.88 | 0.81 – 0.96 | 0.005 |
| 4 | 2,545 (59.62) | 1,724 (40.38) |  | 0.99 | 0.91 – 1.07 | 0.734 | 0.88 | 0.81 – 0.97 | 0.010 |
| 5 (Most Deprived) | 2,267 (60.66) | 1,470 (39.34) |  | 1.03 | 0.95 – 1.12 | 0.496 | 0.86 | 0.78 – 0.95 | 0.004 |

^a^Chi-square P value.

^b^P values in bold are from LRT of the variable’s contribution to the model. Unbolded P values are from a test of whether the OR is different from 1.

^c^Refers to IMD (income domain). For diagnosis year 2012, IMD_2010 was used and for diagnosis years 2013-2017, IMD_2015 was used.

Models are adjusted for: IMD, age, ethnicity, rural/urban residence, government region, stage at diagnosis, receipt of surgery within 6 months, ER status, number of comorbidities, whether discussed at MDT, and diagnosis year (apart from positive HER2+ status definition model).

Abbreviations: ER: Estrogen receptor; HER2: Human epidermal growth factor receptor 2; IMD: Index of Multiple Deprivation; LRT: Likelihood ratio test; MDT: Multi-disciplinary team; OR: Odds ratio; SACT: Systemic anti-cancer therapy; 95% CI: 95% Confidence interval.
