# Supplementary material for: Sociodemographic Disparities in HER2+ Breast Cancer Trastuzumab Receipt: An English Population-Based Study
Source: Cancer Epidemiol Biomarkers Prev. 2024 Jul 15;33(10):1298–310. doi: 10.1158/1055-9965.EPI-24-0144 (PMC7616541; doi:10.1158/1055-9965.EPI-24-0144)
Supplement: Supplementary Table S3 Metastatic Disease Trastuzumab Receipt Sensitivity Analysis — _Clean [file epi-24-0144_suppst3.docx]

**Supplementary Table S3** Metastatic disease trastuzumab receipt: sensitivity analyses

|  |  |  |  | **Unadjusted** | | | **Adjusted** | | |
| --- | --- | --- | --- | --- | --- | --- | --- | --- | --- |
|  | Number (%)  Receiving  Trastuzumab | Number (%) Not Receiving Trastuzumab | P Value^a^ | OR | 95% CI | P Value^b^ | OR | 95% CI | P Value^b^ |
| ***Original Analysis: All Stage IV Disease Diagnosed 2012-2017 (n = 2,369)*** | | |  |  |  |  |  |  |  |
|  |  |  |  |  |  |  |  |  |  |
| **Deprivation^c^** | n = 1,062 (44.83) | n = 1,307 (55.17) | 0.314 |  |  | **0.312** |  |  | **0.225** |
| 1 (Least Deprived) | 224 (46.28) | 260 (53.72) |  | 1.00 | ----- ----- | ------- | 1.00 | ----- ----- | ------- |
| 2 | 215 (44.70) | 266 (55.30) |  | 0.94 | 0.73 – 1.21 | 0.622 | 0.85 | 0.64 – 1.13 | 0.270 |
| 3 | 182 (40.90) | 263 (59.10) |  | 0.80 | 0.62 – 1.04 | 0.099 | 0.75 | 0.56 – 1.00 | 0.052 |
| 4 | 229 (44.47) | 286 (55.53) |  | 0.93 | 0.72 – 1.19 | 0.565 | 0.84 | 0.63 – 1.12 | 0.232 |
| 5 (Most Deprived) | 212 (47.75) | 232 (52.25) |  | 1.06 | 0.82 – 1.37 | 0.655 | 1.00 | 0.74 – 1.37 | 0.977 |
|  |  |  |  |  |  |  |  |  |  |
| ***Sensitivity Analysis 1: Diagnosis Date Post Mandated SACT Submission 01/04/2014 – 31/12/2017 (n = 1,629)^4^*** | | | | | |  |  |  |  |
|  |  |  |  |  |  |  |  |  |  |
| **Deprivation^c^** | n = 800 (49.11) | n = 829 (50.89) | 0.456 |  |  | **0.456** |  |  | ------- |
| 1 (Least Deprived) | 168 (49.12) | 174 (50.88) |  | 1.00 | ----- ----- | ------- | ------ | ----- ----- | ------- |
| 2 | 166 (48.97) | 173 (51.03) |  | 0.99 | 0.74 – 1.34 | 0.968 | ------ | ----- ----- | ------- |
| 3 | 137 (44.92) | 168 (55.08) |  | 0.84 | 0.62 – 1.15 | 0.285 | ------ | ----- ----- | ------- |
| 4 | 173 (50.00) | 173 (50.00) |  | 1.04 | 0.77 – 1.40 | 0.818 | ------ | ----- ----- | ------- |
| 5 (Most Deprived) | 156 (52.53) | 141 (47.47) |  | 1.15 | 0.84 – 1.56 | 0.391 | ------ | ----- ----- | ------- |
|  |  |  |  |  |  |  |  |  |  |
| ***Sensitivity Analysis 2: Positive HER2+ Status Definition (n = 1,792)*** | | |  |  |  |  |  |  |  |
|  |  |  |  |  |  |  |  |  |  |
| **Deprivation^c^** | n = 978 (54.58) | n = 814 (45.42) | 0.544 |  |  | **0.544** |  |  | **0.253** |
| 1 (Least Deprived) | 202 (57.06) | 152 (42.94) |  | 1.00 | ----- ----- | ------- | 1.00 | ----- ----- | ------- |
| 2 | 193 (52.59) | 174 (47.41) |  | 0.83 | 0.62 – 1.12 | 0.228 | 0.69 | 0.49 – 0.98 | 0.036 |
| 3 | 176 (51.61) | 165 (48.39) |  | 0.80 | 0.60 – 1.08 | 0.150 | 0.76 | 0.53 – 1.07 | 0.119 |
| 4 | 217 (55.78) | 172 (44.22) |  | 0.95 | 0.71 – 1.27 | 0.726 | 0.89 | 0.63 – 1.27 | 0.533 |
| 5 (Most Deprived) | 190 (55.72) | 151 (44.28) |  | 0.95 | 0.70 – 1.28 | 0.721 | 0.85 | 0.59 – 1.24 | 0.400 |

^a^Chi-square P value.

^b^P values in bold are from LRT of the variable’s contribution to the model. Unbolded P values are from a test of whether the OR is different from 1.

^c^Refers to IMD (income domain). For diagnosis year 2012, IMD_2010 was used and for diagnosis years 2013-2017, IMD_2015 was used.

^4^Multivariable model has inadequate fit, so is not shown.

Models are adjusted for: IMD, age, ethnicity, rural/urban residence, government region, grade (bar positive HER2+ definition model), stage at diagnosis, grade, receipt of surgery within 6 months (bar positive HER2+ definition model), ER status, number of comorbidities, whether discussed at MDT, and diagnosis year.

Abbreviations: ER: Estrogen receptor; HER2: Human epidermal growth factor receptor 2; IMD: Index of Multiple Deprivation; LRT: Likelihood ratio test; MDT: Multi-disciplinary team; OR: Odds ratio; SACT: Systemic anti-cancer therapy; 95% CI: 95% Confidence interval.
